# Supplementary material for: Truck platooning reshapes greenhouse gas emissions of the integrated vehicle-road infrastructure system
Source: Nat Commun. 2023 Aug 15;14:4495. doi: 10.1038/s41467-023-40116-0 (PMC10427667; doi:10.1038/s41467-023-40116-0)
Supplement: Supplementary file 1 — Supplementary Information [file 41467_2023_40116_MOESM1_ESM.pdf]

# Supplementary Information

*for*

## *Truck platooning reshapes greenhouse gas emissions of the integrated vehicle-road infrastructure system*

Huailei Cheng[1,2], Yuhong Wang[1*], Dan Chong[3], Chao Xia[4], Lijun Sun[2*], Jenny Liu[5], Kun Gao[6], Ruikang Yang[2], Tian Jin[2]

1. Department of Civil and Environmental Engineering. The Hong Kong Polytechnic University, Hong Kong SAR.
2. The Key Laboratory of Road and Traffic Engineering of Ministry of Education, Tongji University, Shanghai, China.
3. School of Management, Shanghai University, Shanghai, China.
4. Shanghai Automotive Wind Tunnel Center, Tongji University, Shanghai, China.
5. Department of Civil, Architectural and Environmental Engineering, Missouri University of Science and Technology, Missouri, USA.
6. Department of Architecture and Civil Engineering, Chalmers University of Technology, Gothenburg, Sweden.

*Corresponding authors (Yuhong Wang, yuhong.wang@polyu.edu.hk; Lijun Sun, ljsun@tongji.edu.cn)

# Supplementary Figures

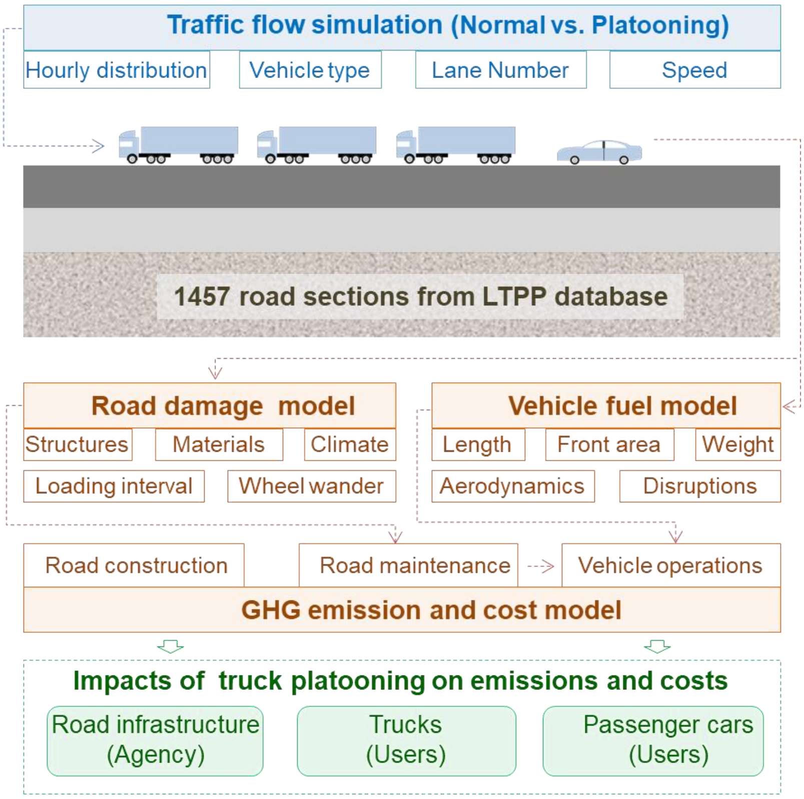


Supplementary Fig. 1. The framework for evaluating the impacts of truck platooning on GHG emissions and cost of the integrated vehicle-road infrastructure system.

a

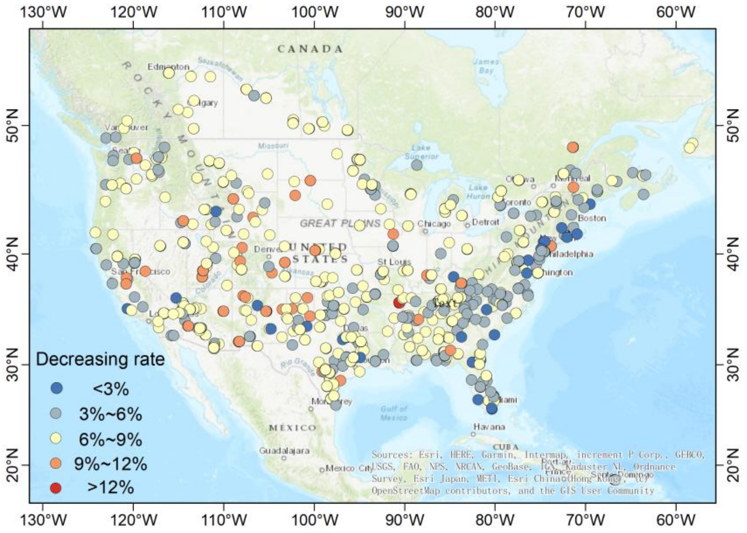

Decreasing rate
<3%
3%~6%
6%~9%
9%~12%
>12%
CANADA
UNITED STATES
GREAT PLAINS
MÉXICO


b

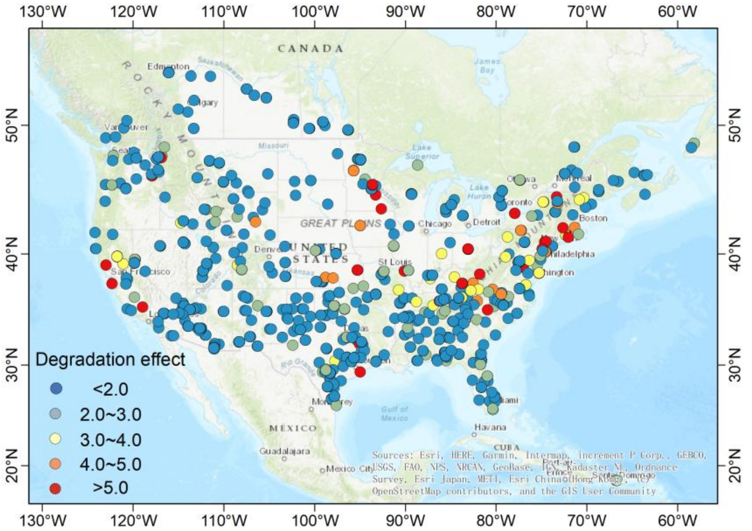

Degradation effect
<2.0
2.0~3.0
3.0~4.0
4.0~5.0
>5.0
CANADA
UNITED STATES
GREAT PLAINS
MÉXICO


c

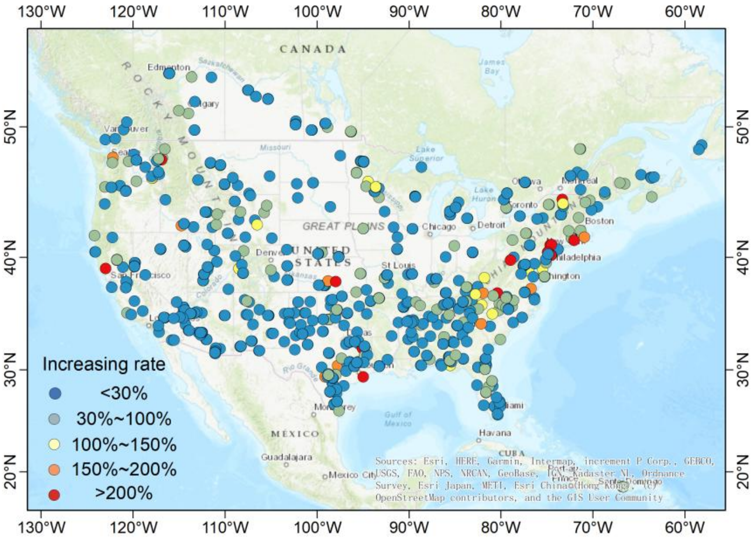

Increasing rate
<30%
30%~100%
100%~150%
150%~200%
>200%
CANADA
UNITED STATES
GREAT PLAINS
MÉXICO

d

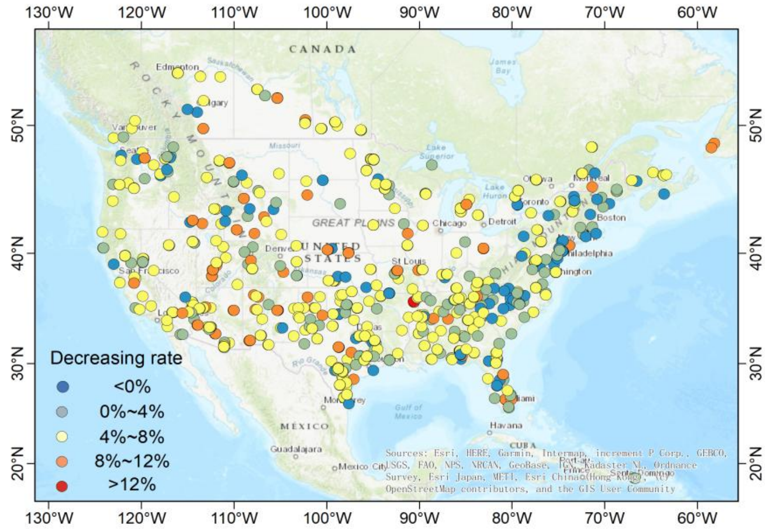


Supplementary Fig. 2. Mapping of different indicators for 1457 road sections. (a), Mapping of the decreasing rates of vehicle emissions. (b), Mapping of degradation effects. (c), Mapping of the increasing rates of road emissions. (d), Mapping of the decreasing rates of the overall vehicle-road emissions. In (a)-(d), some road sections are located close to each other. As a result, the points corresponding to those sections may overlap on the map. The maps were generated using ArcGIS Desktop (version 10.6). Copyright © 1999–2017 Esri Inc. Source data are provided as a Source Data file.

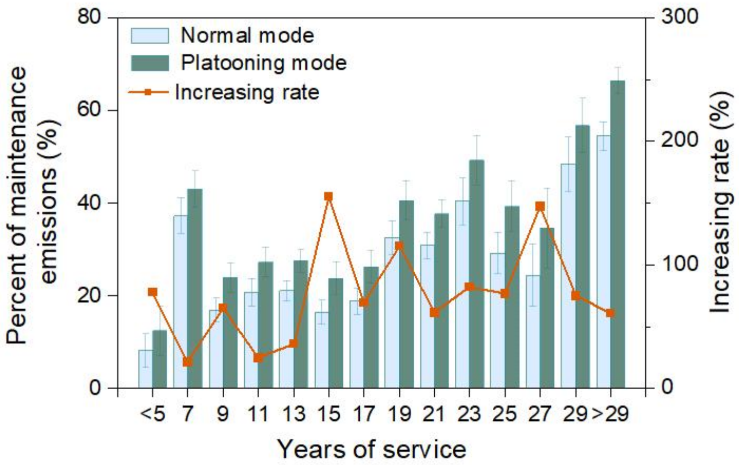


Supplementary Fig. 3. Effects of truck platooning on the road emissions caused by maintenance work. The histogram refers to the percent of maintenance emissions within the overall road emissions (i.e., emissions from the initial construction plus those from the maintenance work) under two operation modes. The line charts refer to increasing rates of maintenance emissions due to platooning. The data in (a) are grouped according to the service lives of the roads. The error bars in (a) refer to ±1.5 times of standard errors (SE) of the mean of each data group. Source data are provided as a Source Data file.

## Supplementary Table

Supplementary Table 1. Variations in vehicle costs and road costs due to truck platooning.

| Indexes | Vehicle costs (User costs) | | | | | | Road costs (Agency costs) | | Overall costs (Agency & User costs) | |
|---|---|---|---|---|---|---|---|---|---|---|
| | Car | | Single truck | | Combo truck | | | | | |
| **Mean**[a] | +3.1% | +13010$ | -4.0% | -14175$ | -3.5% | -2682$ | +20.8% | +20345$ | +4.6% | +16498$ |
| **SE**[b] | 0.3% | 2168$ | 0.1% | 600$ | 0.1% | 161$ | 2.6% | 2050$ | 0.9% | 3597$ |
| **IQR**[c] | 2.8% | 3828$ | 2.0% | 14397$ | 2.5% | 3347$ | 15.4% | 18075$ | 5.8% | 31556$ |

Note: The data shown here represent the variations in annual costs of vehicles/roads generated within a unit length (i.e., 1 kilometer). [a]The mean values are the averaged results derived from 1457 road sections. [b] The SE refers to the Standard Error of each group of data. [c] The IQR refers to the Inter Quartile Range of each group of data.

# Supplementary Methods

## Supplementary Method 1. Acquisitions of road information data based on the LTPP's database

### 1.1 Introduction of the LTPP database

The monitoring data of the road sites, traffic volumes, environmental conditions, road performance, and road maintenance/rehabilitation are obtained from the Long-term Pavement Performance (LTPP) program. The LTPP program, which is initiated by the U.S. Federal Highway Administration (FHWA) and the American Association of State Highway and Transportation Officials (AASHTO), aims to study the performance of in-service roads via both field observations and laboratory tests[1]. It is designed to understand how and why roads perform as they do[2]. To achieve this goal, the LTPP program has monitored, recorded, and accumulated a great amount of road performance-related data since 1987. The data is accessible through a comprehensive information management system (IMS)[2], which consists of a road performance database, LTPP traffic analysis software database, and ancillary information management system archives. The LTPP's road performance database is the main source of data for this study. Data in the database can be downloaded from the website (https://infopave.fhwa.dot.gov/Data/DataSelection).

In-situ road sections located in the U.S. and Canada are chosen and investigated in the LTPP program. Those road sections are designed and constructed by following the standard practices of the highway administration agencies, and they are subject to real-life environmental and traffic conditions. The performance-related data of the LTPP test sections include road structure and construction details, climate conditions, traffic conditions, and road performance. Approximately 280 million records of data have been collected so far, and the number is still climbing. The LTPP's database constitutes the world's largest and most comprehensive road performance database [2].

### 1.2 Road sites under study

A total of 1,457 road sections from the LTPP program are investigated in this study. The sections are selected based on two principles:

1) The road sections are paved with asphalt concrete. This is because the analyzed truck traveling mode (i.e., regular mode or platoon-oriented mode) apparently affects the performance of the asphalt layer due to the visco-elastic nature of asphalt concrete material.

2) Daily traffic data of the chosen road section should be available. This research aims to analyze the potential effects of truck platooning on the performance and Greenhouse Gas (GHG) emissions from road infrastructure. Therefore, traffic data is crucial for analyzing the travel behaviors of the trucks as well as the damage evolution of the road. However, some LTPP road sections lack traffic data, which are thus excluded from the analysis.

The locations of the 1,457 road sections are plotted in Fig. 1- 1. Also shown in the figure is the elevation information. It is seen that the chosen road sections are located at various states in the U.S. and certain provinces in Canada (61 states/districts/provinces and 350 counties). These sections cover all four climate zones classified by FHWA: Dry-Freeze (DF), Wet-Freeze (WF), Dry-Nonfreeze, and Wet-Nonfreeze (WNF)[3, 4]. The wide coverage of these sections makes them representative of the road transportation conditions in North

America.

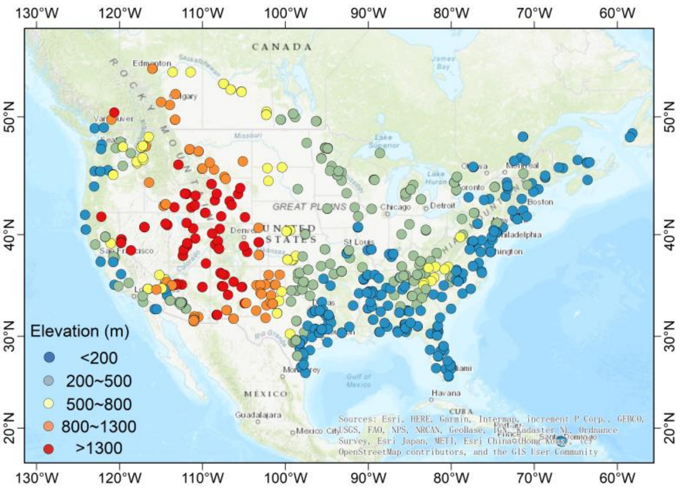


**Fig. 1- 1. The locations of the chosen road sections for this study. Copyright © 1999–2017 Esri Inc.**

Besides geographical locations, the assigned dates (i.e., the dates when the monitoring starts) for the road sections are also extensive. Fig. 1- 2 shows the histogram of the assigned dates of road sections understudy. It is noted that the assigned dates of the sections range widely from 1990.01 to 2020.01. More than 80% of road sections have been monitored since 1990, and this number has increased to 95% since 2000. The above fact reveals that the performance-related data of the roads cover more than 30 years.

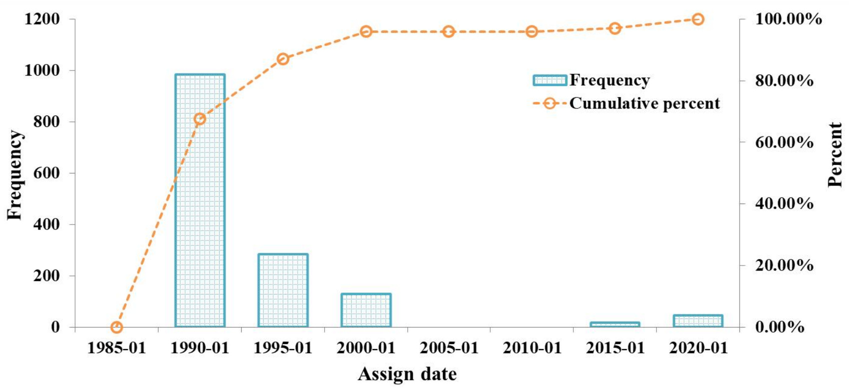


**Fig. 1- 2. The histogram of the assigned dates of road sections**

The selected road sections also cover various functional classifications, which are defined by their roles in connecting traffic. For instance, some roadways are designed to connect the traffic flows between urban areas across the state, and their functions are classified as Urban Principal Arterial – Interstate. The LTPP data contains a total of 12 functional classifications, 11 of which are covered in this study. The distribution of the functional classifications of chosen road sections is summarized in Table 1- 1. Roads with different functional classifications generally experience different levels of traffic and construction/maintenance investment. Therefore, the inclusion of various functional classifications in the research is beneficial in investigating the impacts of truck platooning on different grades of roadways.

**Table 1- 1. The distribution of the functional classifications of chosen road sections.**

| Functional class No | Functional class explanation | Percent (%) |
|---|---|---|
| 1 | Rural Principal Arterial - Interstate | 25.87 |
| 2 | Rural Principal Arterial - Other | 52.09 |
| 6 | Rural Minor Arterial | 10.50 |
| 7 | Rural Major Collector | 2.68 |
| 8 | Rural Minor Collector | 0.62 |
| 9 | Rural Local Collector | 1.03 |
| 11 | Urban Principal Arterial - Interstate | 0.75 |
| 12 | Urban Principal Arterial - Other Freeways or Expressways | 1.10 |
| 14 | Urban Other Principal Arterial | 4.80 |
| 16 | Urban Minor Arterial | 0.48 |
| 17 | Urban Collector | 0.08 |

## 1.3 The structures and materials of the road sections

Information on the structures and materials of the investigated road sections is retrieved from the database. A schematic diagram of the pavement structures and materials is illustrated in Fig. 1- 3. Each road section has its own number of layers, layer types, layer thickness, and materials used for each layer. Material properties such as the modulus and Poisson's ratio of each layer are also needed. Together, the road structure and material properties are used to analyze its mechanical behaviors under the combined effects of truck loads and environmental conditions. Such information is also necessary for calculating GHG emissions associated with road construction.

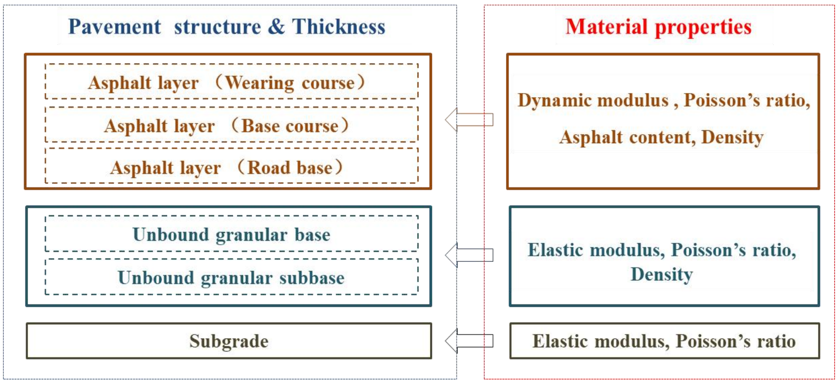


**Fig. 1- 3. The detailed variables determined regarding the structures and material properties.**

An example of road structure data is presented in Table 1- 2. This road section has two construction numbers. Change in the construction number is caused by maintenance or rehabilitation work after the assignment date. For example, Table 1- 2 indicates that this road is overlayed with an additional asphalt layer after initial construction. Variations in construction numbers complicate the road's structural combinations. Finally, a total of 4,019 road structures are evaluated with different construction numbers from the 1,457 road sections.

**Table 1- 2. The exemplary structure data of the road section**

| State code | SHRP ID | Construction No | Layer No | Description | Thickness (cm) | Asphalt content | Density ($g/cm^3$) |
|---|---|---|---|---|---|---|---|
| 1 | 0502 | 1 | 1 | Subgrade | — | — | — |
| 1 | 0502 | 1 | 2 | Subbase Layer | 15.2 | — | 2.15 |
| 1 | 0502 | 1 | 3 | Base Layer | 26.4 | — | 2.15 |
| 1 | 0502 | 1 | 4 | Binder Course Layer | 6.9 | 5.07 | 2.29 |
| 1 | 0502 | 1 | 5 | Original Surface Layer | 2.0 | 5.07 | 2.29 |
| 1 | 0502 | 2 | 1 | Subgrade | — | — | — |
| 1 | 0502 | 2 | 2 | Subbase Layer | 15.2 | — | 2.15 |
| 1 | 0502 | 2 | 3 | Base Layer | 26.4 | — | 2.15 |
| 1 | 0502 | 2 | 4 | Binder Course Layer | 6.9 | 5.07 | 2.42 |
| 1 | 0502 | 2 | 5 | Original Surface Layer | 2.0 | 5.07 | 2.29 |
| 1 | 0502 | 2 | 6 | Overlay | 4.8 | 5.07 | 2.39 |

Asphalt content and density data for the relevant road layers are also shown in Table 1- 2. However, not all the layers in all the road sections have such data. For those layers lacking such data, the average results from the existing asphalt content/density data are used. The stiffness of an asphalt concrete (AC) layer is characterized by its dynamic modulus to reflect the viscoelastic nature of the asphalt material. The LTPP program determines the dynamic modulus of AC using the laboratory modulus test or the prediction model. With the modulus data, a sigmoidal model is used to characterize the modulus master curve of AC based on the following equation:

$$\log(E) = \delta + \frac{\alpha}{1 + e^{\beta - \gamma \log \tau}} \tag{1.1}$$

Where, $E$ is the dynamic modulus (Pa), $\delta$, $\alpha$, $\beta$ and $\gamma$ are the fitting parameters, $\tau$ is the frequency at the reference temperature (Hz), and the frequency at another temperature is calculated by multiplying a shift factor. The model for calculating the shift factor is:

$$\log \alpha_T = \alpha_1 T^2 + \alpha_2 T + \alpha_3 \tag{1.2}$$

Where, $\alpha_T$ is the shift factor, $T$ is the temperature, and $\alpha_1$, $\alpha_2$, $\alpha_3$ are fitting parameters.

The parameters for the sigmoidal models of most AC layers can be found in the LTPP database. For those road sections missing such data, the averaged results are assigned to the AC layers of the sections, as shown in Table 1- 3.

**Table 1- 3. The sigmoidal model parameters for different types of AC layers.**

| Asphalt layer type | $\delta$ | $\alpha$ | $\beta$ | $\gamma$ | $\alpha_1$ | $\alpha_2$ | $\alpha_3$ |
|---|---|---|---|---|---|---|---|
| Surface layer | 3.791 | 2.859 | -1.073 | 0.501 | 0.001 | -0.181 | 3.358 |
| Base course layer | 3.918 | 2.727 | -1.039 | 0.541 | 0.001 | -0.174 | 3.250 |
| Overlay | 3.843 | 2.799 | -0.987 | 0.514 | 0.001 | -0.179 | 3.328 |
| Seal Coat | 3.810 | 2.847 | -1.115 | 0.480 | 0.001 | -0.189 | 3.545 |

| | | | | | | | |
|---|---|---|---|---|---|---|---|
| Surface treatment | 3.350 | 3.447 | -0.843 | 0.377 | 0.002 | -0.221 | 4.023 |

The moduli of other layers including base, subbase, and subgrade layers are characterized using the back-calculated elastic moduli derived from the falling weight deflector (FWD) tests. The FWD tests are conducted on field road pavements, and the results reflect the mechanical responses from the different layers. Similarly, the elastic moduli of some road layers are missing in the database. Therefore, representative modulus values are calculated and shown in Table 1- 4, which are the averaged values of modulus data for such materials. The typical values of Poisson's ratio for different layers are shown in Table 1- 5.

**Table 1- 4. The representative modulus values of the base layer, subbase layer, and subgrade layer.**

| **Layer type** | **Representative modulus (MPa)** |
|---|---|
| Bound (treated) base | 745.5 |
| Bound (treated) subbase | 416.5 |
| Unbound (granular) base | 258.0 |
| Unbound (granular) subbase | 255.6 |
| Subgrade | 253.8 |

**Table 1- 5. The Poisson's ratios of different road layers.**

| **Layer type** | **Poisson's ratio** |
|---|---|
| AC layer | 0.30 |
| Bound (treated) base/subbase layer | 0.30 |
| Unbound (granular) base/subbase layer | 0.35 |
| Subgrade | 0.40 |

## 1.4 The traffic and climatic data of road sections

### 1.4.1 Traffic data

Traffic information in the LTPP database is obtained from the historical data of the sections before assignment or the monitoring data of the section after assignment. The LTPP program has collected the traffic data of the road sections since 1990. In this research, two types of data stored in the traffic module are used: the daily traffic data of the section and the gross vehicle weights (GVW) of the trucks. The daily traffic data is mainly used to simulate the traffic flow on the section under the regular traffic mode and the truck platooning mode. It presents the hourly traffic data on the sections within a day, including the recording hour, the vehicle classification, and the corresponding vehicle number. In LTPP's traffic module, a total of 13 types of vehicles as classified by the FHWA are monitored. More details regarding the hourly traffic data and their applications will be introduced in Supplementary Method 2.

The GVWs of the trucks are used to calculate the road's damage state, which further forms a basis for determining the maintenance activities. In LTPP, weigh-in-motion (WIM) facilities are used to measure representative axle loads on some road sections to calculate GVW. Due to the high costs of installing and operating WIM facilities, however, some sections lack such facilities. For those sections, GVW data from the nearest road sections are assigned. Similar to daily traffic data, the GVW data also covers different vehicle classifications. Fig. 1- 4 plots the

GVWs of one type of single truck (FHWA vehicle classification 5) and one type of combo truck (FHWA vehicle classification 11) on 1,457 road sections for illustration purposes.

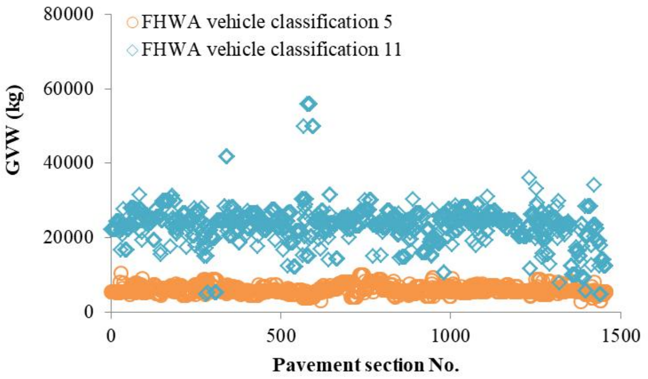


**Fig. 1- 4. The GVWs of two types of vehicles on the 1,457 road sections.**

### 1.4.2 Climatic data

Climate data in the LTPP database include precipitation, temperature, wind, and humidity. Such information is obtained from the U.S. National Climatic Data Center (NCDC) and the Canadian Climate Center (CCC)[1]. The temperature should be paid closer attention to as it predominantly influences the modulus as well as the damage state of the AC layer under traffic load.

In the LTPP program, thermometers are installed in some road sections to monitor the temporal (daily, seasonal, and annual) variations of road temperatures. These field-measured temperature data are extracted from the database to characterize the climatic conditions of the roads. Field measurements suggest that pavement temperature is dependent on its geographical location (i.e., climatic region). Fig. 1- 5 (a) and (b) show the monthly temperature variations at the surface of a road located in Texas, the U.S. and another one in Ontario, Canada, respectively. As expected, temperatures of the road in Texas are higher than those of the road in Ontario. Moreover, the road section in Texas mainly exhibits intermediate- or high-temperatures, with the temperature values all greater than 5°C. However, the road sections in Ontario are subjected to temperatures below 0 °C for about four months. Figs. 1- 5 (c) and (d) further present the histograms of the daily temperature of the two roads.

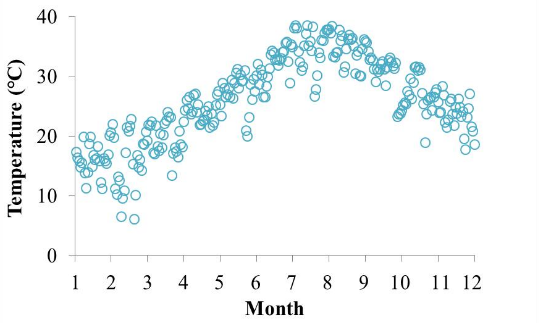


(a)

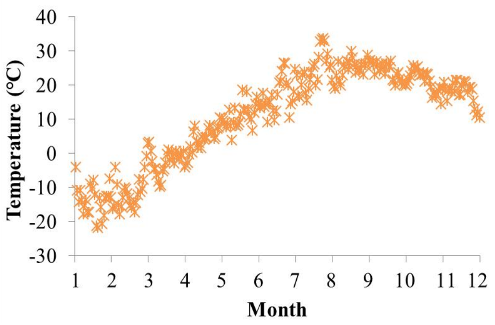


(b)

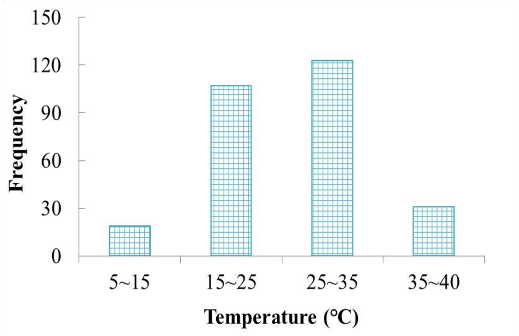


(c)

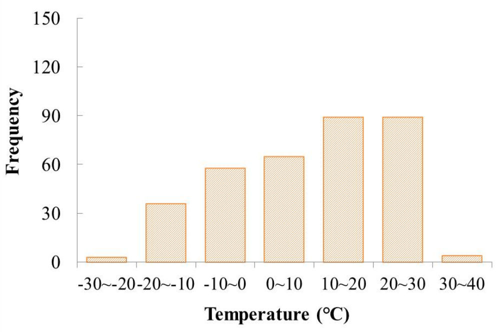


(d)

**Fig. 1- 5. The temperatures at the surface of two roads: (a) the monthly temperature variations of a road in Texas, (b) the monthly temperature variations of a road in Ontario, (c) the histogram of the daily temperature of the road in Texas, and (d) the histogram of the daily temperature of the road in Ontario.**

Besides pavement surface temperature variations associated with different climatic zones, pavement depth also affects temperature. The temperature distributions along the depth profile for the two roads in Texas and Ontario are shown in Fig. 1- 6. Distinguished temperature gradients can be found along the depth profiles of both roads. Therefore, in characterizing the temperatures of different pavement layers, proper temperature gradients are needed.

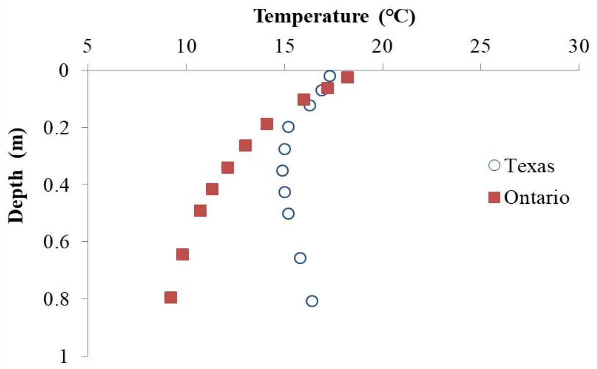


**Fig. 1- 6. Examples of the temperature gradients of two roads in Texas and Ontario.**

The results in Fig. 1- 5 and Fig. 1- 6 indicate that it is essential to assign a proper temperature field (i.e., the temperature distribution in the depth profile) to each road section. In this research, field-measured data are used to assign the temperature field for road sections with monitored temperature data. For the road sections without such data, temperature data from the nearest roads are used, considering that the neighboring roads are subjected to similar environmental conditions. Eventually, temperature fields are assigned to all of the 1,457 road sections. Those temperature data are used to determine the road responses and road damage states under truck loads. The detailed applications of the temperature data will be introduced in Supplementary Method 4.

## 1.5 The performance and maintenance/rehabilitation data of road sections

### 1.5.1 Road performance data

A primary road performance indicator is pavement roughness, which is not only related to riding comfort but also affects fuel consumption. Hence, pavement roughness is evaluated in this research. Pavement roughness is typically represented by an indicator called the International Roughness Index (IRI). Although IRI is extensively monitored in the LTPP

program, there are gaps in the monitoring data. For example, Fig. 1- 7 (a) presents the recorded IRI values of a road section (State Code=1 and SHRP ID=4155) at different service years. IRI data are missing after construction, in the middle of the monitoring period, and at the end of service life. Fig. 1- 7 (a) also indicates that the IRI value drops after road maintenance, but the IRI values immediately before and after the maintenance are also missing. To fill in the missing data, exponential regression models are developed from the existing data, and the missing data are predicted by the models. Fig. 1- 7 (b) illustrates the supplementary IRI data after filling in the missing data. Overall, all the necessary IRI data are well predicted. The complete IRI data is used to calculate the GHG emissions during road operation, and the detailed calculation procedure will be introduced in Supplementary Method 5.

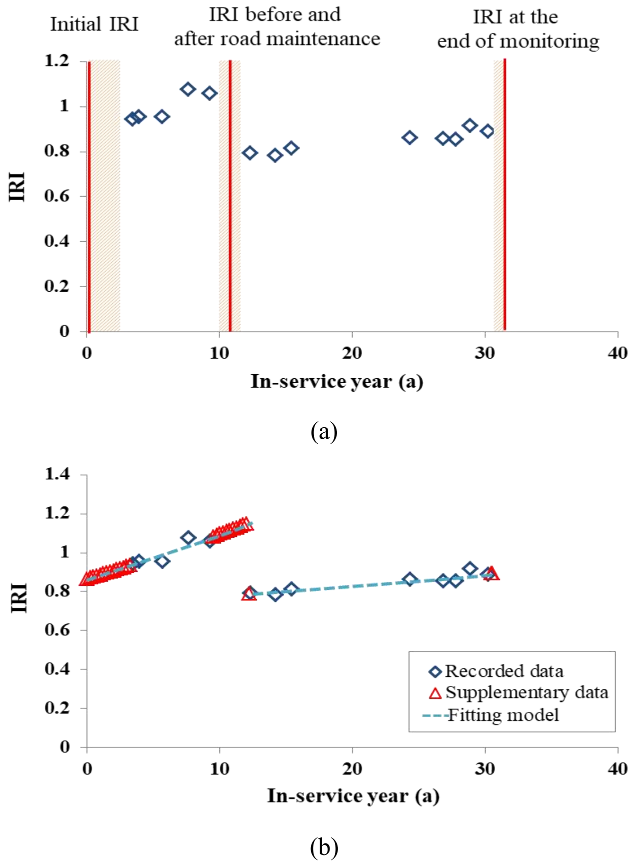


**Fig. 1- 7. The IRI data of the road section: (a) the recorded IRI data, and (b) the IRI data supplemented by the predicted values.**

### 1.5.2 Road maintenance/rehabilitation data

Maintenance or rehabilitation work is frequently conducted to keep the road in good condition. The commonly used maintenance/rehabilitation methods include overlay, milling of existing pavement layers, surface patching, and cracking sealing. However, additional emissions are generated by the vehicle-road infrastructure system due to maintenance/rehabilitation activities and interruption to traffic. Therefore, maintenance/rehabilitation work conducted on a road section is fully considered in analyzing its life-cycle emission. The maintenance/rehabilitation data are extracted from the LTPP database. The types and records of maintenance/rehabilitation activities are summarized in Table 1- 6. With such data, emissions associated with road maintenance/rehabilitation work are calculated. The corresponding calculation procedure will be introduced in Supplementary Method 5.

**Table 1- 6. The maintenance/rehabilitation data collected in this research**

| No | Maintenance/rehabilitation type | Field name |
|---|---|---|
| 1 | Overlay | Date |
| | | Overlay thickness |
| | | Overlay material |
| | | Length |
| 2 | Old layer milling | Date |
| | | Milling thickness |
| | | Length |
| 3 | Surface patching | Date |
| | | Patching reason |
| | | Area of the surface patching |
| | | Depth of the patching |
| | | Patching materials |
| 4 | Crack sealing | Date |
| | | Crack type & severity |
| | | Total length sealed |
| | | Crack sealing materials |

## Supplementary Method 2. Traffic flow simulation of truck platooning

Two types of traffic modes are included for analysis: normal operation and truck platooning. In normal operation, trucks run separately on the road, and adjacent trucks are not intentionally connected. In platooning, a rearward truck intentionally closely follows a preceding truck, and the distance between the adjacent trucks is kept narrow (about 5 m) and stable. Hence, those trucks are closely connected and run in a platoon. Distances between two trucks are not only related to the fuel consumption of trucks, but they also affect the loading mode (i.e., loading interval) on the road pavements and further their durability. We developed a traffic flow simulation framework to determine the spatial-temporal distributions of vehicles under the two traffic modes. The outputs of the simulation are then used to assess road durability as well as the fuel consumption of vehicles. The traffic simulation method is introduced in detail as follows.

### 2.1 The overall framework of traffic simulation

The overall framework of traffic simulation is presented in Fig. 2- 1, which includes three main sections:

- Preparation

The preparation section deals with collecting road and traffic data for simulation and selecting a proper traffic flow model to describe the travel behaviors of vehicles.

- Simulation

In this section, the travel behaviors of vehicles are simulated using the input data and traffic flow model. Both normal operation and the platoon-oriented traffic mode are simulated. These two modes differ in the travel behaviors of trucks and the overall traffic dynamics.

- Results

The result section shows the output data from the simulation, including vehicle ID, travel time and location, speed, and acceleration/deceleration. Based on these outputs, the traffic flow of a road section is generated. The information is used to calculate vehicle emissions and to determine the loading gap between trucks, which is further used to calculate pavement responses and damage accumulation.

### 2.2 Traffic information

As illustrated in Fig. 2- 1, road and traffic information are needed for simulation. The information of a particular road site— road ID, lane width and length, lane number, and speed limit—has been introduced in Supplementary Method 1. More details on traffic information are introduced here.

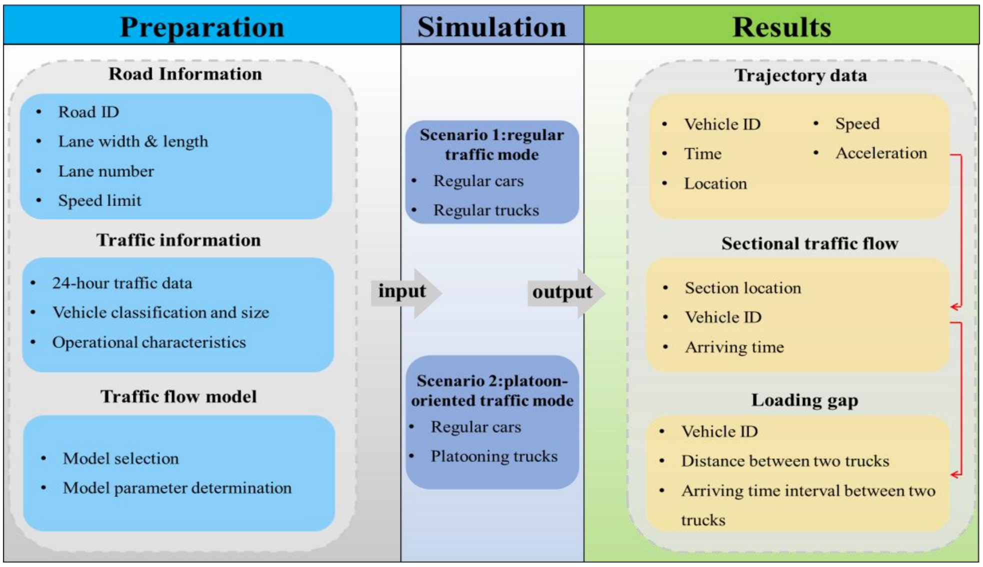


**Fig. 2- 1. The overall framework of traffic simulation.**

Hourly vehicle type and number are recorded in most of the LTPP test sections. The vehicles are classified into 13 classes according to the U.S. Federal Highway Administration (FHWA) definition, as illustrated in Table 2- 1. Because the class 1 vehicle (the motorcycle) is not abundant and has a very small effect on traffic flow, it is not included in traffic simulation. The representative sizes and front areas of those vehicles used for simulation are also presented in Table 2- 1. Vehicle dimensions in Table 2- 1 are based on statistical data collected from truck manufacturers or truck dealers[5-7].

**Table 2- 1. Vehicle information used for simulation.**

| **FHWA classification** | **Representative length (m)** | **Front area ($m^2$)** |
|---|---|---|
| 2. Passenger cars | 4.5 | 7.6 |
| 3. Pickups, panels, vans | 4.8 | 6.6 |
| 4. Heavy buses | 12.0 | 7.8 |
| 5. Single unit 2-axle trucks | 5.8 | 8.7 |
| 6. Single unit 3-axle trucks | 7.3 | 10.7 |
| 7. Single unit 4 or more-axle trucks | 7.3 | 10.7 |
| 8. Single trailer 3- or 4-axle trucks | 11.0 | 10.7 |
| 9. Single trailer 5-axle trucks | 15.2 | 10.7 |
| 10. Single trailer 6 or more-axle trucks | 16.6 | 10.7 |
| 11. Multi trailer 5 or less-axle trucks | 19.0 | 10.7 |
| 12. Multi trailer 6 -axle trucks | 19.0 | 10.7 |
| 13. Multi trailer 7 or more-axle trucks | 23.2 | 10.7 |

Some roads have only daily but not hourly traffic data. For those roads, hourly traffic data are estimated based on the hourly vehicle type distributions collected from other available roads. The hourly average distributions of the 12 types of vehicles are summarized in Table 2- 2. By

inputting the 24-hour traffic data into a simulation loop, the trajectory data of different vehicles on the road can be obtained.

**Table 2- 2. The statistical distribution percentage of 12 types of vehicles within 24h.**

| Hour | Vehicle classification | | | | | | | | | | | |
|---|---|---|---|---|---|---|---|---|---|---|---|---|
| | **2** | **3** | **4** | **5** | **6** | **7** | **8** | **9** | **10** | **11** | **12** | **13** |
| 0 | 0.017 | 0.013 | 0.019 | 0.015 | 0.021 | 0.027 | 0.019 | 0.029 | 0.021 | 0.032 | 0.035 | 0.018 |
| 1 | 0.031 | 0.024 | 0.017 | 0.017 | 0.024 | 0.027 | 0.031 | 0.029 | 0.021 | 0.030 | 0.032 | 0.023 |
| 2 | 0.012 | 0.012 | 0.019 | 0.014 | 0.013 | 0.023 | 0.022 | 0.029 | 0.025 | 0.035 | 0.032 | 0.022 |
| 3 | 0.012 | 0.012 | 0.016 | 0.014 | 0.018 | 0.028 | 0.021 | 0.030 | 0.024 | 0.049 | 0.047 | 0.026 |
| 4 | 0.015 | 0.016 | 0.020 | 0.019 | 0.025 | 0.018 | 0.021 | 0.034 | 0.025 | 0.052 | 0.038 | 0.026 |
| 5 | 0.025 | 0.030 | 0.032 | 0.029 | 0.036 | 0.036 | 0.032 | 0.036 | 0.032 | 0.062 | 0.039 | 0.041 |
| 6 | 0.039 | 0.046 | 0.051 | 0.046 | 0.051 | 0.043 | 0.038 | 0.040 | 0.040 | 0.053 | 0.040 | 0.047 |
| 7 | 0.056 | 0.054 | 0.062 | 0.055 | 0.061 | 0.063 | 0.055 | 0.044 | 0.051 | 0.051 | 0.047 | 0.047 |
| 8 | 0.051 | 0.054 | 0.066 | 0.057 | 0.071 | 0.080 | 0.060 | 0.049 | 0.058 | 0.050 | 0.050 | 0.056 |
| 9 | 0.049 | 0.054 | 0.062 | 0.057 | 0.071 | 0.077 | 0.062 | 0.053 | 0.060 | 0.038 | 0.053 | 0.062 |
| 10 | 0.055 | 0.056 | 0.051 | 0.058 | 0.066 | 0.076 | 0.060 | 0.054 | 0.066 | 0.036 | 0.047 | 0.064 |
| 11 | 0.055 | 0.057 | 0.054 | 0.060 | 0.071 | 0.070 | 0.066 | 0.055 | 0.071 | 0.045 | 0.042 | 0.079 |
| 12 | 0.054 | 0.056 | 0.066 | 0.063 | 0.073 | 0.078 | 0.070 | 0.055 | 0.072 | 0.036 | 0.042 | 0.073 |
| 13 | 0.061 | 0.059 | 0.074 | 0.065 | 0.072 | 0.091 | 0.068 | 0.056 | 0.073 | 0.040 | 0.045 | 0.080 |
| 14 | 0.057 | 0.062 | 0.077 | 0.068 | 0.064 | 0.048 | 0.060 | 0.054 | 0.064 | 0.034 | 0.042 | 0.070 |
| 15 | 0.059 | 0.064 | 0.063 | 0.069 | 0.072 | 0.081 | 0.053 | 0.052 | 0.063 | 0.034 | 0.074 | 0.066 |
| 16 | 0.065 | 0.068 | 0.051 | 0.068 | 0.055 | 0.053 | 0.054 | 0.051 | 0.055 | 0.042 | 0.056 | 0.049 |
| 17 | 0.067 | 0.064 | 0.054 | 0.061 | 0.036 | 0.028 | 0.054 | 0.049 | 0.045 | 0.035 | 0.033 | 0.035 |
| 18 | 0.061 | 0.055 | 0.042 | 0.048 | 0.029 | 0.012 | 0.045 | 0.045 | 0.039 | 0.036 | 0.033 | 0.026 |
| 19 | 0.046 | 0.045 | 0.028 | 0.039 | 0.018 | 0.008 | 0.030 | 0.040 | 0.026 | 0.024 | 0.032 | 0.025 |
| 20 | 0.039 | 0.036 | 0.026 | 0.029 | 0.017 | 0.014 | 0.029 | 0.035 | 0.019 | 0.031 | 0.034 | 0.022 |
| 21 | 0.032 | 0.029 | 0.021 | 0.022 | 0.016 | 0.009 | 0.022 | 0.031 | 0.020 | 0.045 | 0.036 | 0.021 |
| 22 | 0.024 | 0.020 | 0.016 | 0.016 | 0.010 | 0.005 | 0.015 | 0.028 | 0.017 | 0.057 | 0.034 | 0.013 |
| 23 | 0.017 | 0.013 | 0.014 | 0.011 | 0.010 | 0.007 | 0.013 | 0.025 | 0.011 | 0.050 | 0.037 | 0.009 |

## 2.3 Traffic flow models

The traffic flow models describing the normal operation and truck platooning are introduced as follows.

### 2.3.1 The model for normal operation

An improved Intelligent Driver Model (IDM) is used to characterize the behaviors of trucks and cars in normal operation. The IDM, initially proposed by Treiber *et al.* [8], is widely used in microscopic traffic simulation. Later, Ye and Zhang[9] found that the impact of the car-truck interaction on traffic flow is significant. They proposed that four types of car-truck interactions need to be taken into account in simulating the heterogeneous car-truck traffic flow: car-following-car (CC), car-following-truck (CT), truck-following-car (TC), and truck-following-truck (TT). To consider the heterogeneity of traffic flow, an improved IDM is further proposed by Treiber *et al.* [10], as expressed Equation (2.1).

$$\begin{cases} \dfrac{d^2x_n(t)}{dt^2} = a_n\left[1-\left(\dfrac{v_n(t)}{V_n}\right)^{\delta_n} - \left(\dfrac{S_n\left(v_n(t),\Delta v_n(t)\right)}{\Delta x_n(t)-L_n}\right)^2\right] \\ S_n\left(v_n(t),\Delta v_n(t)\right) = s_{n,0}+s_{n,1}\cdot\sqrt{\dfrac{v_n(t)}{V_n}}+\tau_n v_n(t) - \dfrac{v_n(t)\cdot\Delta v_n(t)}{2\sqrt{a_n b_n}} \end{cases} \tag{2.1}$$

Where, $a$ is the maximum acceleration, $v$ is the actual velocity, $V$ is the desired velocity, $\delta$ is the acceleration exponent, $S(\cdot)$ is the desired minimum gap, $s_{n,0}$ and $s_{n,1}$ are the jam distances, $\tau$ is the safe time headway, $b$ is the desired deceleration, $L$ is the

leading vehicle length, and $\Delta v_n(t)$ is the velocity difference between the vehicle $n$ and its preceding vehicle $n-1$, which is calculated using Equation (2.2).

$$\Delta v_n(t) = v_{n-1}(t) - v_n(t) \tag{2.2}$$

Each parameter (i.e., $a$, $\delta$, $V$, $s_0$, $s_1$, $\tau$ and $b$) has four alternatives. For instance, $a$ can be $a_{CC}$, $a_{CT}$, $a_{TC}$ and $a_{TT}$, which correspond to the accelerations of four types of car-truck combinations, respectively. The leading vehicle length $L$ has two alternatives, namely $L_c$ and $L_t$, which correspond to the lengths of the car and truck, respectively.

The input parameters for the improved IDM are summarized in Table 2- 3. Those parameter values are calibrated by using the Next Generation SIMulation (NGSIM) trajectory data with high time resolution[11]. Specifically, the vehicle trajectory data collected at Hollywood Freeway (U.S.101) and Berkeley Highway (I-80) in California are used for calibration. Consequently, the adopted parameters are regarded to reflect the realistic traffic flow characteristics of the U.S. highway.

**Table 2- 3. The input parameters for the improved IDM[11].**

| Variables | Car-truck combinations | | | |
|---|---|---|---|---|
| | CC | CT | TC | TT |
| $a$ (m/s$^2$) | 1.01 | 1.03 | 0.78 | 0.74 |
| $V$ (m/s) | 27.0 | 19.3 | 20.6 | 17.7 |
| $s_0$ (m) | 0.85 | 1.35 | 1.11 | 1.53 |
| $s_1$ (m) | 0.19 | 0.27 | 0.12 | 0.36 |
| $\tau$ (s) | 1.2 | 1.4 | 1.8 | 2.0 |
| $b$ (m/s$^2$) | 2.26 | 2.12 | 1.70 | 1.61 |
| $\delta$ | 4 | 4 | 4 | 4 |

### 2.3.2 The model for truck platooning

The platooning trucks in the model are operated with two sub-controllers that are designed for different control objectives[12]:

- A cruising controller to maintain a user-set desired speed if a preceding vehicle is absent.
- A gap-regulating controller to maintain a constant time gap with its predecessor in car-following situations.

**(1) Cruising controller**

The control objective of the cruising mode is to maintain the user-desired speed when the preceding vehicle is absent or far away. The acceleration of a cruising vehicle is modeled as:

$$a_{n,k} = k_0 \cdot \left(v_{set} - v_{n,k-1}\right) \tag{2.3}$$

Where, the control gain $k_0$ is a parameter to determine the rate of speed error for acceleration, $v_{set}$ is the driver's desired speed and $v_{n,k-1}$ is the speed of vehicle $n$ at time step $k$. The value of $k_0$ is assumed as 0.4 s$^{-1}$ according to reference[13].

**(2) Gap-regulating mode**

In the gap-regulating mode, the car-following response of the first truck in the platoon is

described by:

$$a_{n,k} = k_1 \cdot e_{n,k} + k_2 \cdot \left(v_{n-1,k-1} - v_{n,k-1}\right) \quad (2.4)$$

Where, $e_{n,k}$ is the gap error of vehicle $n$ at time step $k$. An existing study found that the vehicle acceleration depends on the gap error and the speed difference with the preceding vehicle, where their feedback gains $k_1$ and $k_2$ are 0.23 $s^{-2}$ and 0.07 $s^{-1}$, respectively[14].

For the following trucks in the platoon, their speeds are calculated by the speed in a previous time step $v_{n,k-1}$, the gap error $e_{n,k-1}$ in a previous time step and the corresponding derivative. The following form is used for calculation.

$$v_{n,k} = v_{n,k-1} + k_p \cdot e_{n,k-1} + k_d \cdot \dot{e}_{n,k} \quad (2.5)$$

Where, $k_p$ and $k_d$ are determined as 0.45 $s^{-1}$ and 0.25, respectively[14, 15].

The gap error $e_{n,k-1}$ is calculated by using the following form:

$$e_{n,k-1} = x_{n-1.k-1} - x_{n.k-1} - L - t_{des} \cdot v_{n,k-1} - d_0 \quad (2.6)$$

$$d_0 = \begin{cases} 0, & v \geq 10m/s \\ -0.125v + 1.25, & v < 10m/s \end{cases} \quad (2.7)$$

Where, $x_{n-1.k-1} - x_{n.k-1}$ is the inter-truck spacing, $t_{des}$ is the desired time gap, $L$ is the truck length, and $d_0$ is the spacing margin[16].

The other model parameters for traffic flow simulation are listed in the following table.

**Table 2- 4. The additional parameters of the model for traffic flow simulation.**

| **Parameters** | **Values** |
|---|---|
| Number of trucks in a platoon | Three trucks |
| Desired time gap | 0.9 s |
| Maximum acceleration | 2 $m/s^2$ |
| Maximum deceleration | -4 $m/s^2$ |

## 2.4 Simulation loop and outputs

Based on the input data and the traffic flow models, the behaviors of independent trucks or the trucks in a platoon are simulated. For each simulation case, one hour is spent on warm-up. After the warm-up period, different classes of vehicles are generated for simulation. The total number of the generated vehicles depends on the input traffic volume. An additional module is implemented in the simulation loop to guide vehicle generation. This module ensures that the hourly number of vehicles passing through a road section in the simulation matches the recorded traffic data.

After the simulation, the trajectory data (including vehicle ID, traveling time, location, speed, acceleration/deceleration) of each vehicle are recorded. The traffic flow for each road section is generated and used to determine the loading gap distribution between different trucks. The typical patterns of vehicle trajectories in the two traffic modes are illustrated in Fig. 2- 2, where the blue lines represent the trajectories of cars, and the red lines represent the

trajectories of trucks. Fig. 2- 2 indicates that the red lines (i.e., trucks) distribute evenly under normal operation, implying that the trucks generally operate freely and independently. However, the red lines in platooning tend to agglomerate due to the trucks running close to each other. In platooning, gaps between adjacent trucks are relatively small, causing the trucks' trajectory lines to overlap.

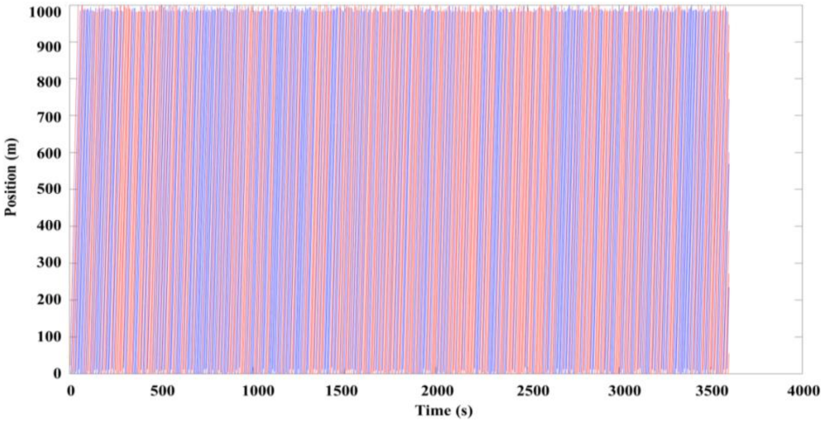


(a)

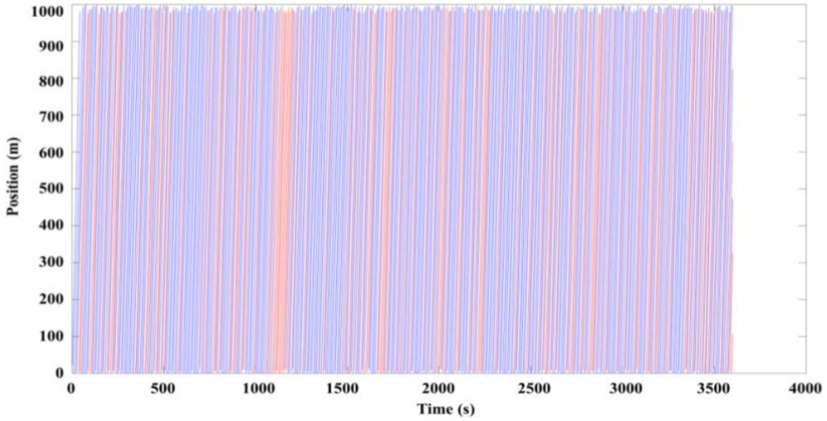


(b)

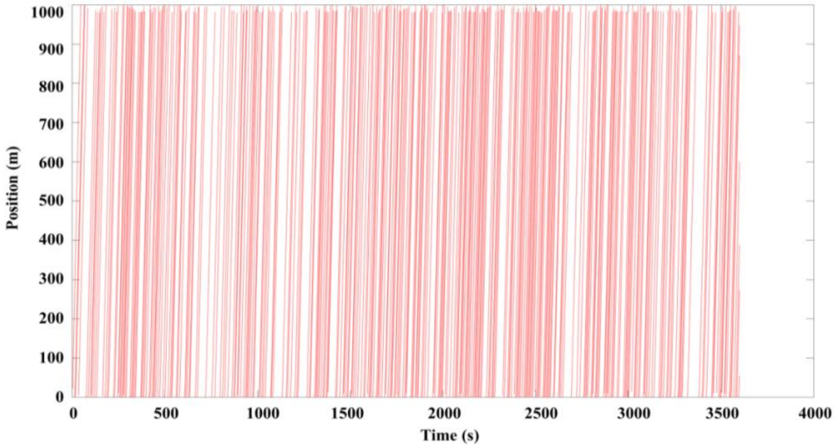


(c)

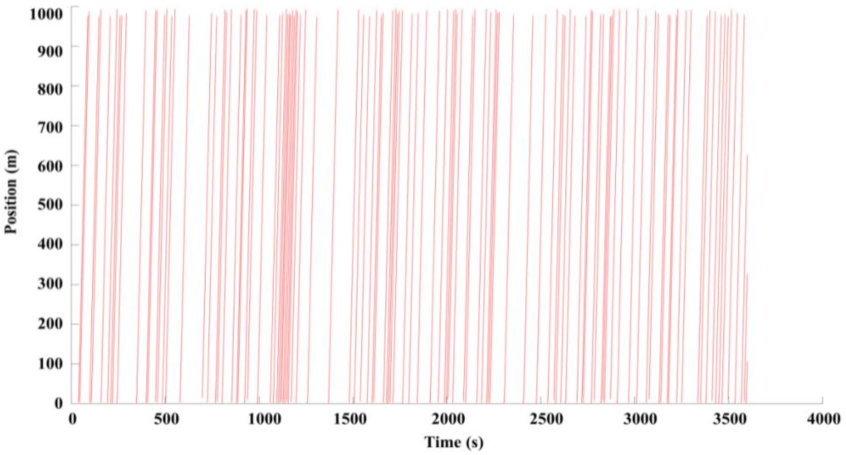


(d)

**Fig. 2- 2. The vehicle trajectories: (a) the trajectories of both cars and trucks in normal operation, (b) the trajectories of both cars and trucks in platooning, (c) the trajectories of trucks in normal operation, and (d) the trajectories of trucks in platooning.**

## Supplementary Method 3. Fuel consumption model for truck platooning

The fuel consumption of the vehicle is directly related to the GHG emission of the vehicle-road infrastructure system. In this research, a fuel consumption model is developed and used to evaluate the fuel economy of the vehicle in a platoon or not. Based on the fuel consumption, the emission during vehicle operation is assessed. The detailed procedures for developing the fuel consumption model are introduced as follows.

### 3.1 Aerodynamics of truck platooning

The aerodynamics of trucks running in platooning mode differ from those of an independent truck due to the drag-reduction effects from the lead truck, which has to overcome most of the drag resistance while the remaining trucks in the platoon are subject to much less air resistance[17-19]. The reductions in drag resistances of the non-lead trucks help lower the required work from truck engines, thereby decreasing the overall fuel consumption of a truck platoon[20-23]. The fuel-saving potential of a truck platoon is highly dependent on the drag resistance it has to overcome. Therefore, to assess the fuel economy of a truck platoon, it is necessary to investigate the aerodynamics of the trucks in the platoon.

Computational Fluid Dynamics (CFD) simulation has been commonly applied to evaluate the aerodynamics of vehicles [24]. This research also used the CFD simulation tool to investigate the truck platoon's aerodynamic behaviors. The simulation cases consist of three types of trucks with different dimensions and axle configurations, four types of separation distances, and five types of truck numbers in a platoon. These combinations enable us to evaluate the aerodynamics of different forms of truck platoons comprehensively. The schematic diagrams of the three types of trucks assessed in this research are shown in Fig. 3- 1.

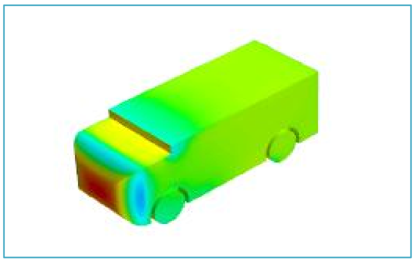

(a)

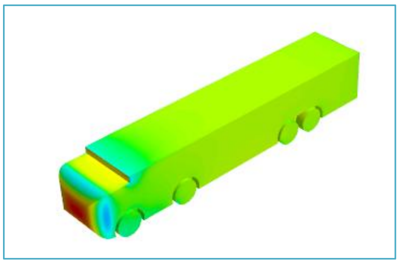

(b)

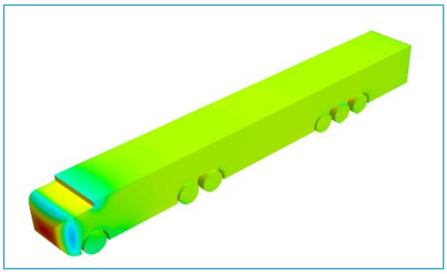

(c)

**Fig. 3- 1. The schematic diagrams of three types of trucks evaluated in simulation: (a) two-axle truck with a small dimension (5.8m×2.2m×1.9m), (b) four-axle truck with an intermediate dimension (11m×2.2m×1.9m), and (c) six-axle truck with a large dimension (16.6m×2.2m×1.9m).**

The numerical models in CFD simulation are developed based on the adopted truck configurations, and the 1/8 scale truck model is used to save computation time. The overall computational domain was determined as 6H×16L×10W, as shown in Fig. 3-2. The symbols H, L, and W in the figure refer to the height, length, and width of the truck model, respectively. The boundary conditions of the computational domain are determined as follows:

The inlet and outlet are the velocity inlet and the pressure outlet, respectively. The truck body and the stationary ground are set as the non-slip wall conditions, while the moving wall has the same velocity as the inlet. The rest of the boundaries use symmetric boundary conditions.

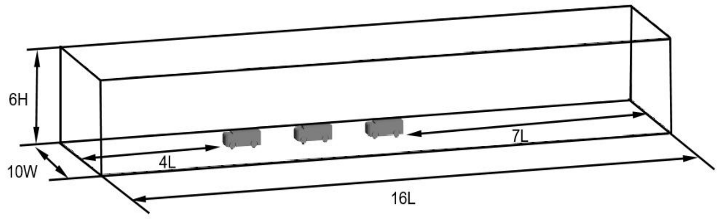


**Fig. 3- 2. The schematic diagram of the overall computational domain.**

As for the meshing strategy, the mixed grids are assigned for the computation domain. The prism layers are used for the wall boundaries, whilst the hexahedral grids are used for the rest of the domain. The trimming technique is applied to transit the areas between the prism layers and the hexahedral grids. To improve the computing efficiency, fine mesh is used around the truck, while relatively coarse mesh is used for the area far away from the truck. Specifically, the mesh size for the prism layer is 1 mm, while that at the truck surface is 5 mm.

Based on simulations, the aerodynamics of the trucks running separately or in a platoon are determined and compared. Example comparison results are presented in Fig. 3- 3, where the calculation results of two-axle trucks are used for illustration. As shown in Fig. 3- 3 (a) and (b), the air pressure coefficients of the independent truck differ from those of the trucks in a platoon. Clearly, the pressure coefficients of the middle truck or trailing truck in the platoon are lower than those of an individual truck, indicating that platooning reduces the air resistance of the non-lead trucks. Similar results can be observed for other simulation cases. They are not shown here for the interest of brevity.

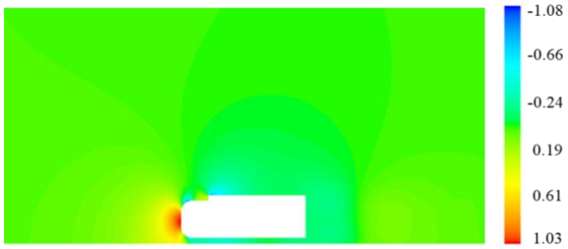


(a)

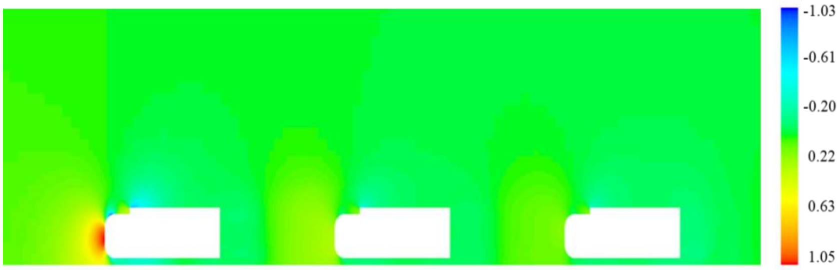


(b)

**Fig. 3- 3. The comparisons of air pressure coefficients of (a) an independent truck and (b) the platooned trucks.**

Based on the simulation results, the drag coefficients (defined as $C_D$ ) for the three independent trucks are calculated and compared, as shown in Fig. 3- 4. As expected, the

$C_D$ increases with the truck size. A truck with a large dimension is subjected to more air resistance and a higher drag coefficient than a small truck. The drag coefficients of the trucks located at different positions of a platoon (i.e., lead position, middle position, trailing position) are illustrated in Fig. 3- 5. In this typical case, the platoon consists of three successive two-axle trucks. The separation distance between two adjacent trucks is 0.2 $L$ ($L$ = truck length). Fig. 3- 5 indicates that $C_D$ of the lead truck is the highest, followed by the middle truck and then by the trailing truck. Not only the middle truck and trailing truck in the platoon experience less air resistance as compared with the lead truck, but the $C_D$ of the lead truck (0.359) is lower than that of the independent truck (0.450 as shown in Fig. 3- 4). Therefore, platooning can reduce the drag coefficients of the entire fleet. This finding also applies to platooning configurations consisting of the different truck types (i.e., four-axle truck and six-axle truck) or truck numbers (e.g., four trucks in a platoon or five trucks in a platoon).

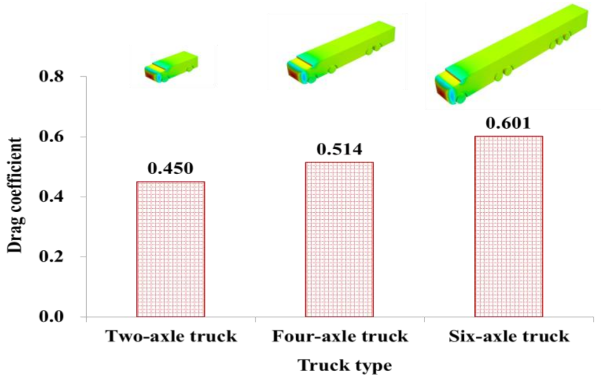


**Fig. 3- 4. The comparisons of drag coefficients of three types of independent trucks.**

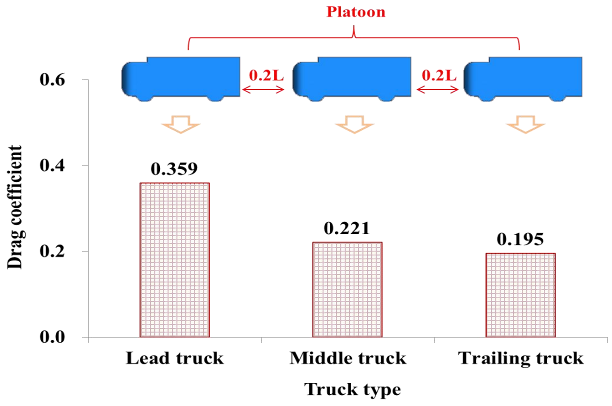


**Fig. 3- 5. The drag coefficients of the trucks located at different positions of a platoon.**

The reduction in drag coefficient caused by platooning is characterized by the drag coefficient saving rate ($\Delta C_D$). $\Delta C_D$ is calculated as the percentage of the saved drag coefficient to the original one. It is found that $\Delta C_D$ is very sensitive to the separation space between trucks and truck length but less affected by the truck numbers of a platoon. Example trends of $\Delta C_D$ with separation space and truck length are presented in Fig. 3- 6 (a) and (b), respectively. Apparently, $\Delta C_D$ decreases with the separation space and increases with the truck length. Using all the data, a model was developed to estimate $\Delta C_D$ for trucks in a platoon, as shown in Equation (3.1).

$$\Delta C_D = [a \cdot \ln(S) + b] \cdot L^c \tag{3.1}$$

Where, $S$ is the separation distance, $L$ is the truck length, and $a$, $b$ and $c$ are the fitting coefficients which are summarized in Table 3- 1.

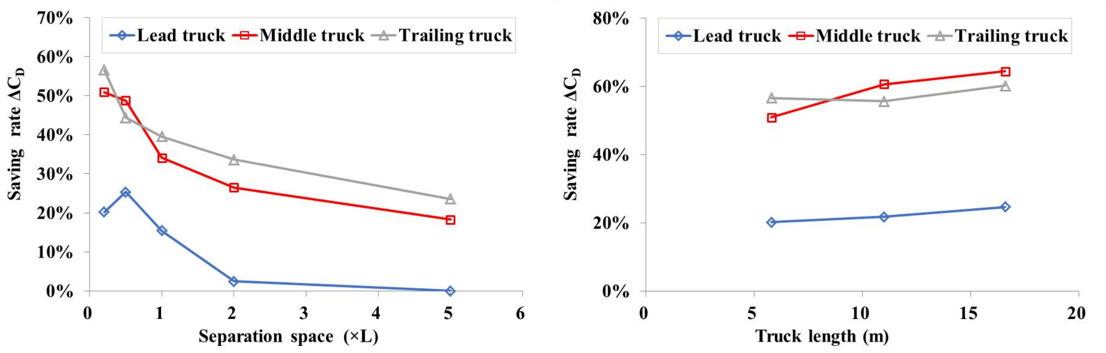


(a) (b)

**Fig. 3- 6. Example trends of $\Delta C_D$ with: (a) separation space and (b) truck length.**

**Table 3- 1. Fitting coefficients of parameters *a*, *b*, and *c*.**

| **Position of a truck in a platoon** | ***a*** | ***b*** | ***c*** | $R^2$ |
|---|---|---|---|---|
| Head | -0.022 | 0.095 | 0.503 | 0.842 |
| Middle | -0.044 | 0.236 | 0.462 | 0.980 |
| Trailing | -0.045 | 0.301 | 0.328 | 0.971 |

To further illustrate the accuracy of the developed model, the calculated $\Delta C_D$ values from Equations (3.1) are compared with the measured ones in Fig. 3- 7. As can be observed, the developed model can well estimate the $\Delta C_D$ of the trucks in a platoon.

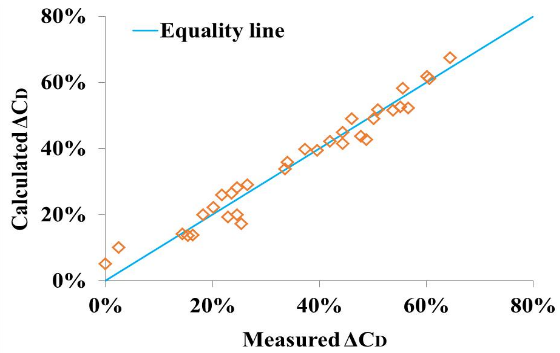


**Fig. 3- 7. Comparisons between the calculated and measured $\Delta C_D$ values.**

## 3.2 Prediction of fuel consumption savings for trucks in a platoon

Section 3.1 reveals that the drag coefficients of the trucks in a platoon are lower than those of independent trucks. Drag coefficient is one of the main factors affecting a truck's fuel consumption. Consequently, trucks in a platoon would consume less fuel than those independent trucks. In this section, models for predicting the fuel consumption of different truck configurations will be developed. The models will turn the effect of drag coefficient reduction into fuel saving.

An operating truck is subjected to several types of resistance, including inertia, rolling resistance, and air resistance [25-27]. Inertia is caused by the acceleration or deceleration of the

truck, while the rolling and air resistances come from the road and the air-fluid around the truck, respectively. The requested engine power to overcome the resistances can be expressed by the following equation:

$$P = \eta v(F_r + D) + I = \eta v(r_0 mg + \frac{1}{2}\rho v^2 AC_D) + I \tag{3.2}$$

Where, $P$ is the engine power, $\eta$ is the efficiency coefficient, $F_r$ is the rolling resistance, $D$ is the air resistance, $I$ refers to the inertia-resistance-related component, $r_0$ is the coefficient of road resistance, $m$ is the mass of the truck, $\rho$ is the density of the air, $v$ is the velocity of the truck relative to the air-fluid, and $A$ is the front area of the truck.

The fuel consumption of a truck can be calculated based on its engine power using:

$$FC \equiv bsfc[P] \tag{3.3}$$

Where, $FC$ is the fuel consumption, $bsfc$ is the brake-specific-fuel-consumption of the engine. Based on this form, the saving rate of the truck's fuel consumption ( $\Delta FC$ ) in a platoon is calculated as follows:

$$\Delta FC = \frac{FC_0 - FC_1}{FC_0} \tag{3.4}$$

Where, $FC_0$ is the fuel consumption of a separately running truck, and $FC_1$ is the fuel consumption of the truck in a platoon.

Substituting Equation (3.2) and Equation (3.3) into Equation (3.4) results in the following:

$$\Delta FC = \frac{\frac{1}{2}\rho v^2 A(C_{D0} - C_{D1})}{r_0 mg + \frac{1}{2}\rho v^2 AC_{D0}} \tag{3.5}$$

Where, $C_{D0}$ is the air drag coefficient of a separately running truck that can be predicted by CFD simulation results, and $C_{D1}$ is the air drag coefficient of the truck in a platoon.

In Equation (3.5), the component ( $C_{D0} - C_{D1}$ ) can be calculated as follows:

$$C_{D0} - C_{D1} = \frac{C_{D0} - C_{D1}}{C_{D0}} \cdot C_{D0} = \Delta C_D \cdot C_{D0} \tag{3.6}$$

Where, $\Delta C_D$ is the saving rate of the drag coefficient that can be calculated based on Equation (3.1).

Combining Equations (3.5) and (3.6), the final form for calculating the fuel-saving rate for a truck in a platoon is derived as:

$$\Delta FC = \frac{[a \cdot \ln(S) + b] \cdot L^c}{1 + \frac{r_0 mg}{\frac{1}{2}\rho v^2 A \cdot (0.014 \cdot L + 0.366)}} \tag{3.7}$$

Where, $a$ , $b$ and $c$ refer to the fitting coefficients which are shown in Table 3- 1.

### 3.3 Calibration of the fuel consumption model

Models developed in Section 3.2 are based on the truck's aerodynamics obtained from CFD simulations. In real situations, a truck's fuel consumption is also influenced by other factors such as engine temperature, lateral offset, and environmental factors such as heat and humidity. As these factors also play a role in fuel consumption, errors may arise if they are

excluded in fuel consumption prediction. Hence, Equation (3.7) needs to be calibrated via field-measured data that reflect those influencing factors. The data used for calibration comes from the Partially Automated Truck Platooning (PATP) project sponsored by the FHWA[21, 28]. This project tested the fuel economy of a three-vehicle truck platooning system in the field. The test was conducted in accordance with the SAE J1321 Fuel Consumption Test Procedure[29]. Specifically, a portable tank filled with fuel was installed on the truck and weighed before the trip. After the trip, the weight of the tank was measured again, and the fuel consumption of the trucks was accurately determined. Fuel consumption of independent trucks and platooned ones were compared. The information on the truck, road, and air conditions used in the PATP project are summarized in Table 3- 2.

**Table 3- 2. The parameters of the truck, road, and air conditions in the PATP project[28].**

| Parameters | Units | Values |
|---|---|---|
| Truck mass ($m$) | kg | 29400 |
| Trucks peed ($v$) | km/h | 105 |
| Front area of the truck ($A$) | $m^2$ | 10.7 |
| Drag coefficient ($C_D$) | / | 0.57 |
| Air density ( $\rho$ ) | $kg/m^3$ | 1.2 |
| The coefficient of road resistance ( $r_0$ ) | / | 0.006 |

By substituting the parameters in Table 3- 2 into Equation (3.7), the fuel saving rates of truck platooning were predicted. The predicted fuel savings were then compared with the measured ones to determine the calibration factor for Equation (3.7). The derived calibration factor (Defined as $M$ ) is fitted using Equation (3.8). The final fuel prediction model after calibration is obtained and shown as Equation (3.9).

$$M = 0.433 \cdot e^{0.008 \cdot S} \qquad (R^2=0.996) \tag{3.8}$$

$$\begin{aligned}\Delta FC &= \frac{M \cdot [a \cdot \ln(S) + b] \cdot L^c}{1 + \dfrac{r_0 mg}{\frac{1}{2}\rho v^2 A \cdot (0.014 \cdot L + 0.366)}} \\ &= \frac{0.433 \cdot e^{0.008 \cdot S} \cdot [a \cdot \ln(S) + b] \cdot L^c}{1 + \dfrac{r_0 mg}{\frac{1}{2}\rho v^2 A \cdot (0.014 \cdot L + 0.366)}}\end{aligned} \tag{3.9}$$

Where, $S$ is the separation distance, $L$ is the truck length, $r_0$ is the coefficient of road resistance, $m$ is the mass of the truck, $\rho$ is the density of the air, $v$ is the velocity of the truck relative to the air fluid, $A$ is the front area of the truck, and $a$ , $b$ and $c$ are the fitting coefficients which have been summarized in Table 3-1.

The fuel savings predicted with the updated model are further compared with the measured ones in Fig. 3- 8. Clearly, the predicted fuel savings of the platoon fit very well with the field-measured ones. As a result, the calibrated model (Equation (3.9)) is reliable enough to estimate the actual fuel savings of the platooning trucks.

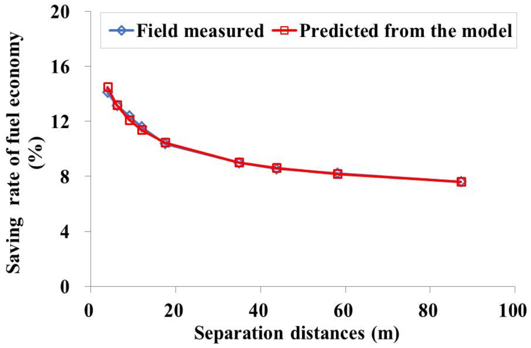


**Fig. 3- 8. The predicted fuel savings versus the field-measured ones.**

As mentioned in Section 2.2, 10 different types of trucks or heavy buses are included in this research. The representative lengths ( $L$ ) and front areas ( $A$ ) for those trucks have been presented in Table 2- 1. Based on the data in Table 2- 1 and Equation (3.9), the fuel-saving rate of a truck in a platoon can be calculated, and the fuel consumption of the truck can be calculated using the following model:

$$FC_P = FC_0 \cdot (1 - \Delta FC) \quad (3.10)$$

Where, $FC_P$ is the fuel consumption of the truck in a platoon, $FC_0$ is the fuel consumption of the separately running truck, and $\Delta FC$ is the saving rate of the truck's fuel consumption due to platooning.

The fuel consumption of a separately running truck (i.e., $FC_0$ ) is evaluated by referring to the statistical Motor-Vehicle Travel (VMT) data from FHWA, as summarized in Table 3- 3[30]. It is noted that the average miles per gallon of fuel (MPG) for different types of trucks are recorded in the table. With those MPG data, the fuel consumption of an independent truck is determined. Passenger cars are assumed to have the same fuel economy under normal traffic and truck-platooning modes, considering that the aerodynamic behaviors of passenger cars on highways are much less affected by truck platooning.

**Table 3- 3. The statistical Motor-Vehicle Travel (VMT) data from FHWA[30].**

| Year | Items | Light-duty vehicles | Single trucks | Combo trucks |
|---|---|---|---|---|
| 2019 | Number of motor vehicles registered | 253,814,184 | 10,160,433 | 2,925,210 |
| 2019 | Average miles traveled per vehicle | 11,520 | 12,278 | 59,929 |
| 2019 | Person-mile of travel (millions) | 4,894,385 | 124,746 | 175,305 |
| 2019 | Average fuel consumption per vehicle (gallons) | 518 | 1,639 | 9,909 |
| 2019 | Average miles traveled per gallon of fuel consumed | 22.2 | 7.5 | 6.0 |

## Supplementary Method 4. Damage model for road section under truck platooning

Road pavement is continuously damaged by repetitive truck loads during its service life. As a result, it needs to be periodically maintained or even rehabilitated. The maintenance or rehabilitation work such as sealing the cracks, patching the holes, or milling the old roads, generates GHG emissions. In evaluating the life-cycle emission of road, it is essential to consider those maintenance/rehabilitation activities. The maintenance/rehabilitation period for the road pavement is dependent on its damage state. An elevated damage level demands more frequent maintenance or rehabilitation.

Commonly observed damages on road pavements can be divided into two groups: permanent deformation (also known as rutting) and cracking-related distresses (fatigue cracking, potholes, etc.). The LTPP database suggests that cracking-related distress is the predominant type of pavement failure. Therefore, this research focuses on cracking-related distresses only. The damage state assessment of asphalt pavement is complicated due to the fatigue-healing behaviors of asphalt concrete (AC) layers. Damage accumulation within an AC layer is influenced by the time gap (i.e., rest period) between adjacent loads[31-34]. A longer rest period promotes a more appreciable healing effect on the asphalt layer and thus results in less damage accumulation. In the truck platooning mode, however, the rearward truck follows the preceding truck closely, and the distance between the adjacent trucks is very narrow (roughly 5m). Consequently, the rest periods between trucks in the platooning mode are quite different from those experienced during a normal operation. Differences in the rest period lead to different pavement damage accumulation processes, which further influence the maintenance/rehabilitation frequencies and the overall emissions from the road. This chapter aims to introduce a damage model for road pavement under different truck operation conditions. With the developed model, the damage state of a road pavement under the normal or platooning truck loading mode can be determined. The information is further used to assist in scheduling maintenance/rehabilitation activities and calculating the corresponding emissions.

### 4.1 Laboratory and field loading tests

#### 4.1.1 Laboratory loading tests

Both laboratory and field loading tests were conducted in this research to simulate the damage effects of truck loads on road pavements. The applied laboratory test is called the four-point bending (4PB) fatigue loading test. Three types of loading waveforms were used in the 4PB fatigue tests. They correspond to the strain waves in road pavements induced by single-axle, tandem-axle and tridem-axle configurations of a truck, respectively. The schematic diagram of the applied loading waves is shown in Fig. 4- 1.

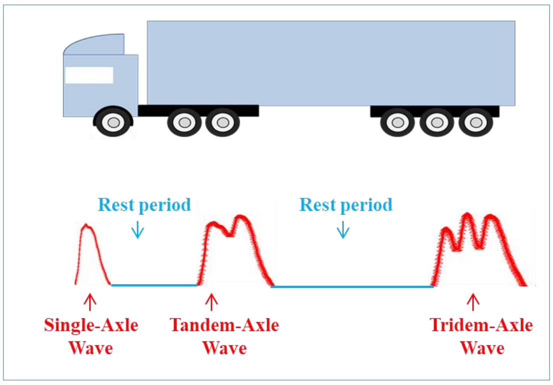


**Fig. 4- 1. The schematic diagram of the applied loading waves.**

A total of three temperature levels (20°C, 10°C and 0°C) were considered in 4PB tests to simulate different environmental conditions. At each test temperature, three different strain levels were applied. The various strain levels were used to characterize the influences of truck weights on road damage. In addition, the rest period (time interval between two adjacent axle loadings) is introduced in the 4PB test to investigate the healing effect of the rest period on the asphalt concrete's damage (Fig. 4- 1). The applied rest period includes four magnitudes: 0s, 0.1s, 0.5s, and 1s. For each loading scenario, two to six parallel fatigue tests were conducted to control test variability. The photographs of the apparatus and specimens in the 4PB loading test are presented in Fig. 4- 2.

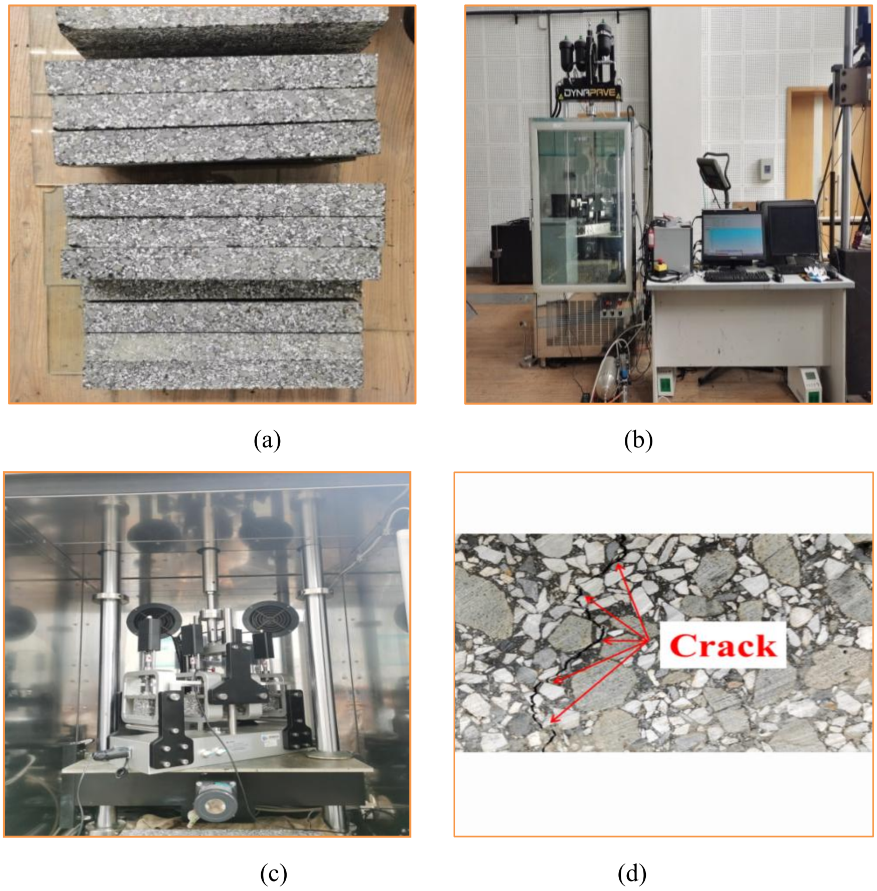


**Fig. 4- 2. The apparatus and specimens in the 4PB loading test: (a) the specimens, (b) overall view of the loading apparatus and the data acquisition system, (c) inside view of the loading apparatus, and (d) damage state (crack) of the specimen after the test.**

Based on the 4PB test results, the fatigue lives of AC samples under various strain waves, rest periods, and temperatures were analyzed and compared. The typical comparison results are shown in Fig. 4- 3. The fatigue lives of the AC samples vary with strain levels approximately in a linear mode on the log-log scale, regardless of loading waveforms. The specimen's fatigue life is reduced with an increase in strain level. At the same strain level, the fatigue life of the specimen subject to the single-axle wave is higher than that subject to the tandem-axle wave, and the fatigue life associated with the tridem-axle wave is the lowest. The results indicate that loading waveforms induced by the multi-axles result in much lower fatigue lives of the samples than those of samples subject to single-axle load. This is because the multi-axle waveform has multi pulse peaks with a longer loading duration. It can thus be concluded that the multi-axle loading waveform inflicts more fatigue damage to the AC sample than the single-axle waveform does.

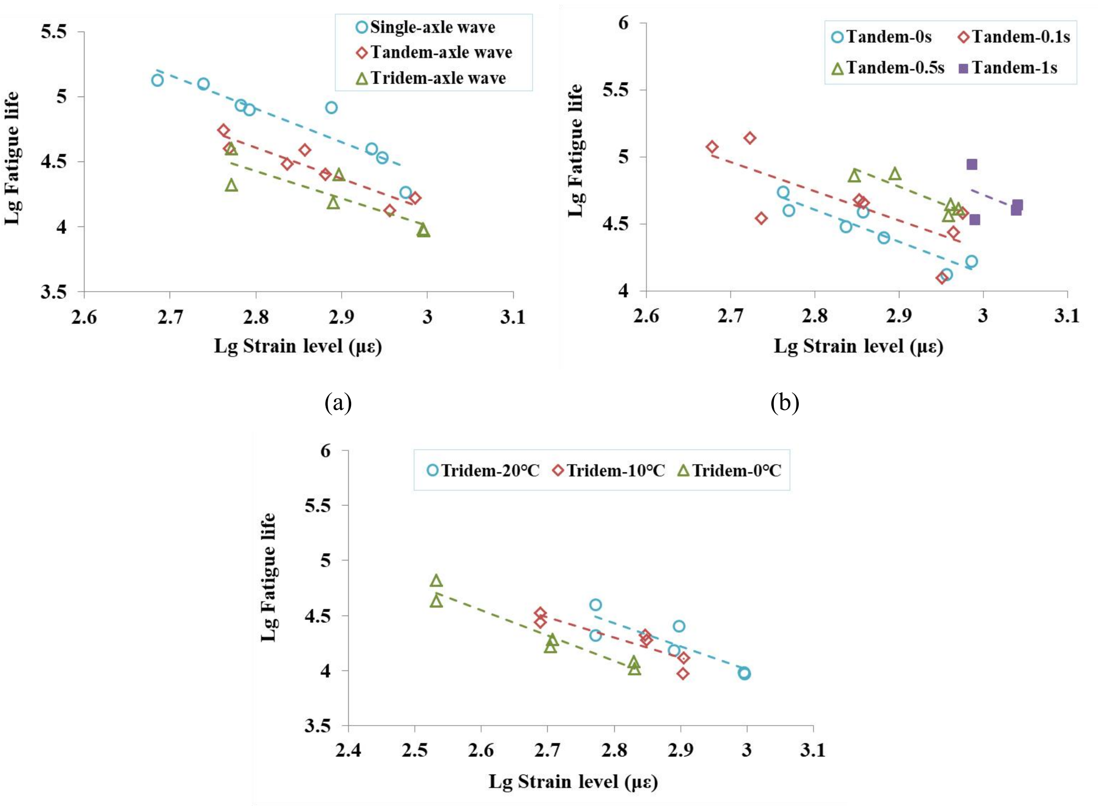


(a) (b)

(c)

**Fig. 4- 3. The comparisons of fatigue lives of AC samples at (a) three loading waveforms, (b) four rest periods, and (c) three temperatures.**

At the same strain level, the fatigue lives measured with rest periods (0.1s, 0.5s, 1s) are apparently higher than those measured with no rest period (0s), as shown in Fig. 4- 3(b). This phenomenon has been proven in previous studies[35-39]. The increase in fatigue life with a longer rest period is attributed to its healing effects on AC material. With the rest period, AC can resist more fatigue loading repetitions as a portion of fatigue damage can be healed. A longer rest period leads to a longer fatigue life for the specimen, but the fatigue life cannot be extended indefinitely with the rising rest period. In addition, the fatigue lines (the plots of fatigue life versus strain) at different rest periods are approximately parallel to each other, suggesting that the effects of strain level on the fatigue life are nearly unaffected by rest period level. As indicated in Fig. 4- 3(c), temperature also has an apparent effect on the AC's

fatigue life, irrespective of loading waveforms. Higher temperature is associated with a longer fatigue life of the specimen if the strain level is kept the same. The slopes of the Lg (fatigue life) versus Lg (strain level) lines are similar at different temperatures. This implies that the impacts of the strain levels on the fatigue life are almost unaffected by the test temperature levels.

Two advanced analysis methods namely the dissipated energy (DE) method and the viscoelastic continuum damage (VECD) method were further used to process the fatigue test results. The relationships between the initial DE (IDE) and the fatigue life of AC material obtained at different rest periods and temperatures are presented in Fig. 4-4. It is seen that the fatigue life of AC varies approximately linearly with IDE on the log-log scale. An increase in IDE is associated with a reduction in fatigue life, and the trends are the same for the three types of loading waveforms. This indicates that the IDE vs. fatigue life relationships tend to be unaffected by the waveform.

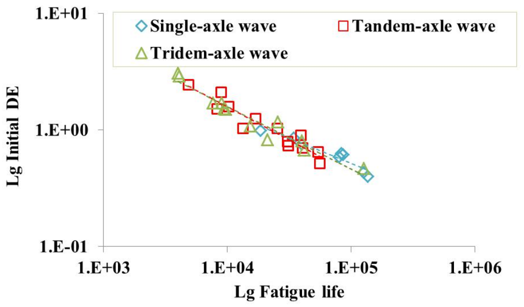


(a)

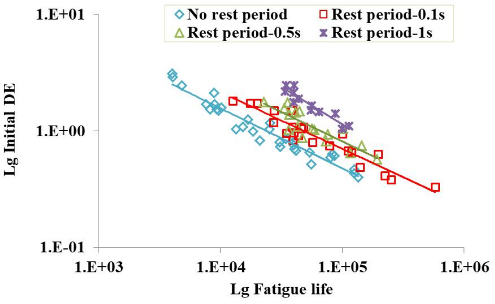


(b)

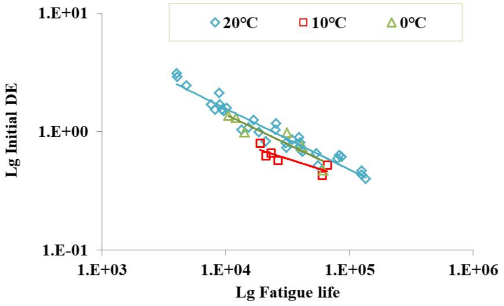


(c)

**Fig. 4- 4. The IDE vs. fatigue life relationships obtained under (a) different strain waves, (b) different rest periods and (c) different temperatures.**

Fig. 4-4 (b) shows that IDE-fatigue life relationships depend on rest periods. Clearly, as the rest period increases, the IDE-fatigue life line shifts up and right almost parallelly. This implies that, at the same level of IDE, a specimen's fatigue life will be extended with the rest period being increased. The healing effect associated with the different rest periods apparently contributes to the prolonged fatigue life of a specimen under the same level of IDE. However, the effect of temperature on the IDE-fatigue life relationships is not so obvious, as seen in Fig. 4-4 (c). The IDE-fatigue life line at 20°C coincides with that at 0°C, and both are located slightly above that at 10°C.

In the VECD method, the pseudo stiffness named *C* and the damage parameter named *S* are applied to quantify the fatigue damage evolution of a material [40-43]. The *C*-*S* curves obtained at different strain waveforms and rest periods are presented in Fig 4-5. It can be observed that

the *C*-*S* curves obtained at different strain levels generally collapse with each other, which supports the common observation that the *C*-*S* curve of AC is free from the effect of loading amplitude. However, there are obvious differences among the *C*-*S* curves derived from the three types of loading waveforms, as shown in Fig. 4-5(a). The *C*-*S* curve induced by the single-axle wave is located at the top position, followed by the one corresponding to the tandem-axle wave and then by the one corresponding to the tridem-axle wave. Such positions are consistent with the fact that the single-axle wave is associated with higher fatigue life than the tandem-axle or tridem-axle waves.

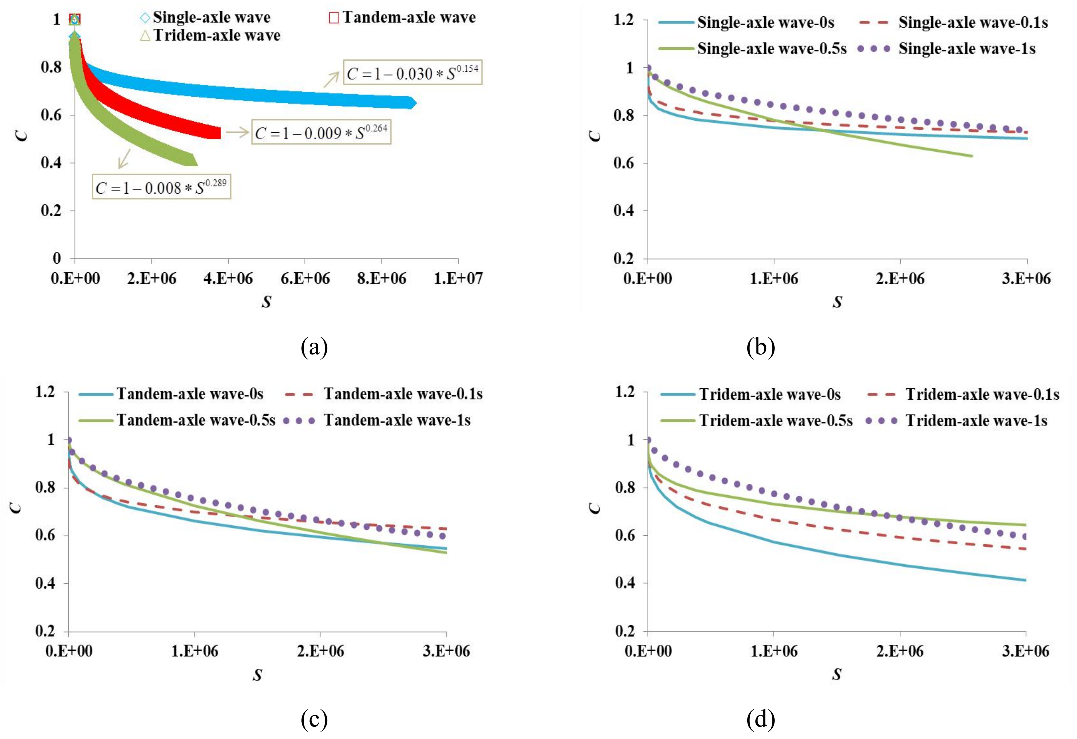


**Fig. 4- 5. The *C*-*S* curves obtained under (a) different strain waveforms, and (b)~(d) different rest periods.**

As shown in Figs. 4-5(b)~(d), the rest period has a remarkable effect on the C-S curve of the AC, regardless of strain wave type. Generally, the curves obtained from the tests with rest periods are positioned above that without rest period (i.e., rest period=0s), especially at the initial stage of the test. This phenomenon reflects the fact that the damage evolution of a specimen slows down with the available rest period, agreeable with the previous observations that the rest period helps heal the mixture from damage and prolongs its fatigue life[34, 37, 38].

From the above discussions, it can be concluded that strain level, loading waveform, rest period, and temperature all have noticeable effects on a specimen's fatigue life. The fatigue life increases with a decreasing strain level, a decreasing axle number, an increasing rest period, and an increasing temperature. Therefore, all these influencing factors are included in developing the fatigue life prediction model for AC. In addition, the initial stiffness modulus of AC is included in the model, to take into account the impact of AC's nature on its fatigue behaviors. Ultimately, the following function was developed to predict the fatigue life of AC material under different loading scenarios.

$$N_f = a \cdot \left(\frac{1}{\varepsilon}\right)^b \cdot \left(\frac{1}{E}\right)^c \cdot e^{(d \cdot T + f \cdot RP)} \tag{4.1}$$

Where, $N_f$ is the fatigue life, $\varepsilon$ is the strain level (με), $E$ is the initial stiffness modulus (MPa), $T$ is the temperature (°C), $RP$ is the rest period (s), and $a$, $b$, $c$, $d$, $f$ are fitting parameters.

The fitted model parameters with different strain waveforms are summarized in Table 4- 1. The predicted fatigue lives based on the model are compared with the measured ones in Fig. 4-6. It can be seen that the developed fatigue life prediction model well characterizes the relationships between fatigue life and different influencing factors. The correlation coefficients ($R^2$) for the models concerning tandem-axle wave and tridem-axle wave exceed 0.8, while that for the model concerning single-axle wave is relatively low at 0.723.

**Table 4- 1. Fitting results of fatigue life prediction models.**

| Strain wave | *a* | *b* | *c* | *d* | *f* | $R^2$ |
|---|---|---|---|---|---|---|
| Single-axle wave | $8.593\times10^{16}$ | 2.769 | 1.230 | 0.046 | 1.062 | 0.723 |
| Tandem-axle wave | $2.823\times10^{14}$ | 2.353 | 0.974 | 0.043 | 1.360 | 0.823 |
| Tridem-axle wave | $8.891\times10^{13}$ | 2.051 | 1.115 | 0.042 | 1.310 | 0.866 |

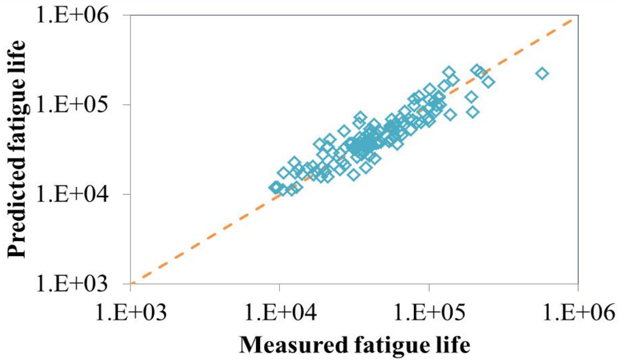


**Fig. 4- 6. Comparisons between the measured and estimated fatigue life.**

### 4.1.2 Field loading tests for calibration

While the test and analysis results above indicate that the fatigue life of an AC sample prepared and tested in laboratory conditions can be well predicted, the fatigue behaviors of AC layer in field pavements can be quite complicated due to variations in actual dimensions and stress states. Therefore, laboratory models need to be calibrated. Full-scale pavement loading tests were used to calibrate the laboratory fatigue models[44]. The full-scale tests were conducted on a field road pavement with a large loading facility named MLS 66. The MLS 66, as presented in Fig. 4-7, is the most advanced truck-load simulator. Based on this powerful facility, a total of 1,000,000 field loading repetitions were applied on the road pavement to simulate the effects of truck loads. During the tests, the damage states of the field road pavements were continuously monitored using the portable seismic property analyzer (PSPA). AC samples were also extracted from the field road pavements and assessed by laboratory tests. By comparing the field measurements and the laboratory test results, the shift factor (*SF*) between the field and the laboratory conditions was determined to be 1.382, with field fatigue life for AC layer being larger than laboratory one. With this shift factor, the model as presented in Equations (4.1) is adjusted to describe the fatigue behaviors of the field AC layer. The calibrated prediction model is shown as follows.

$$N_f = a \cdot \left(\frac{1}{\varepsilon}\right)^b \cdot (\frac{1}{E})^c \cdot e^{(d \cdot T + f \cdot RP)} \cdot SF \quad (4.2)$$

Where, $N_f$ is the fatigue life, $\varepsilon$ is the strain level (με), $E$ is the initial stiffness modulus (MPa), $T$ is the temperature (°C), $RP$ is the rest period (s), and $SF$ is the shift factor which has a value of 1.382.

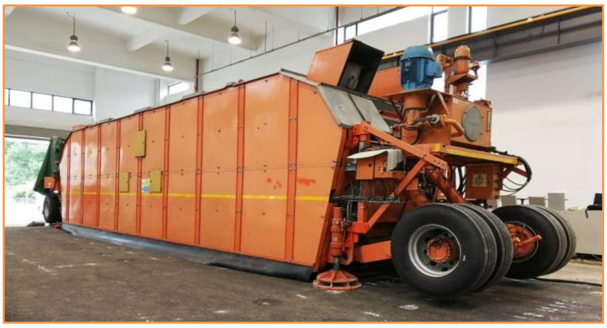

**Fig. 4- 7. The MLS 66 loading facility.**

## 4.2 Road response prediction model

As discussed in Section 4.1, the fatigue life of an AC material is highly dependent on load-induced strain levels, along with other influencing factors. Therefore, the strain response of field pavement needs to be determined. In this research, the pavement's strain response is calculated based on the multi-layer elastic (MLE) theory, which has been widely used in road pavement design specifications including the U.S. mechanistic-empirical pavement design guide (MEPDG) and China's specification[45, 46]. The overall framework for pavement response calculation is presented in Fig. 4- 8.

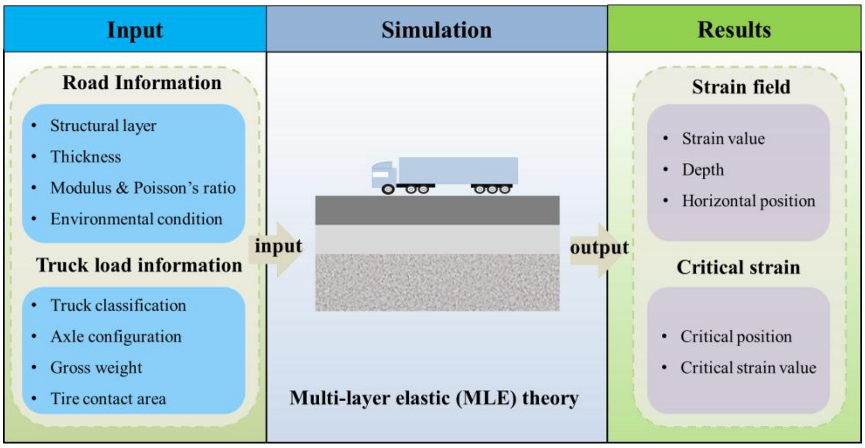


**Fig. 4- 8. The overall framework for road response calculation.**

As shown in Fig. 4- 8, information on road and truck load is needed as input data for pavement response calculation. Road information includes road structures (arrangement of structural layers & thickness), material properties (modulus & Poisson's ratio), and environmental conditions (temperature). These data have been introduced in Supplementary Method 1. Truck load information includes truck classification, axle configuration, gross weight and tire contact area, which has been introduced in Section 1.2. The spatial-temporal distributions of trucks under normal traffic mode and truck-platooning mode have been determined in Section 2.4. With the above input data, the strain field in a road under truck

loading is calculated using the MLE theory. The strains at different depths and horizontal positions (i.e., the relative positions to the loading area) are calculated to determine the critical position with the maximum strain. The evaluated pavement positions regarding different axle configurations are presented in Fig. 4- 9.

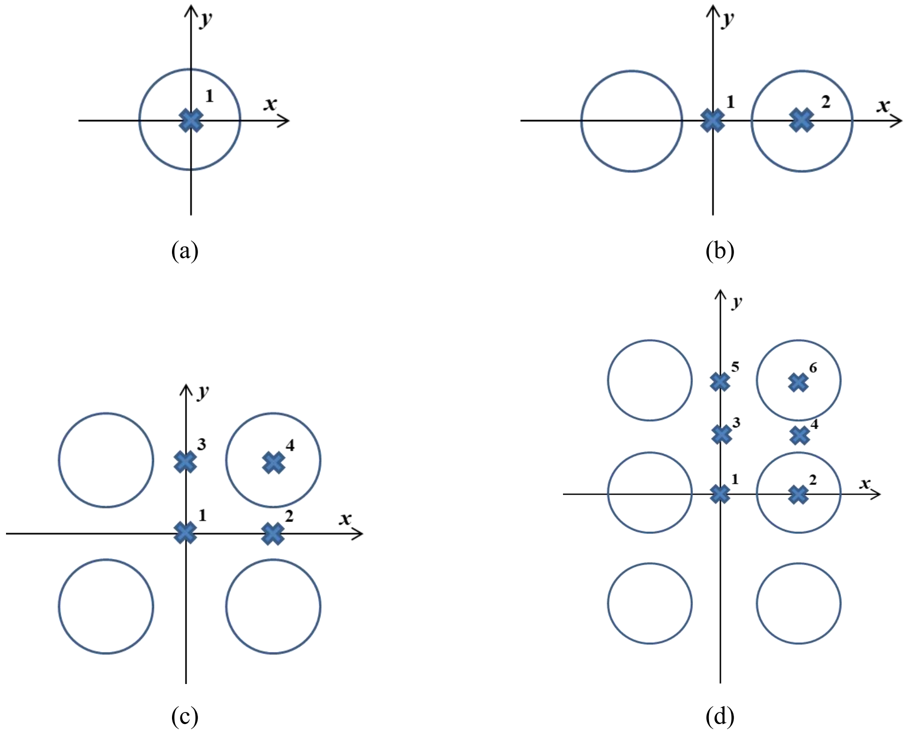


**Fig. 4- 9. The evaluated positions regarding different axle configurations: (a) single-axle with a single tire, (b) single-axle with a dual tire, (c) tandem-axle with dual tires, and (d) tridem-axle with dual tires.**

Wheel wander, which refers to the lateral offset of a vehicle from the wheel path center, is also an important factor that influences the critical strain of the pavement. The lateral offsets of non-platooning trucks (i.e., human-driven trucks) generally follow a normal distribution, as reported in the NCHRP's research[46]. On the other hand, platooning trucks can have their lateral offsets more precisely designed, thanks to the superior control ability of platooning technology. Existing studies have proposed several wheel wander modes for platooning trucks to reduce the damaging impacts of truck loads on the pavement [47, 48]. In this research, we considered two of these modes:

(1) Platooning mode 1: Platooning trucks are controlled to load evenly on three sub-lanes of the wheel path to lower the critical strain at the wheel center.

(2) Platooning mode 2: Platooning trucks are controlled to load evenly across the wheel path to distribute the truck damage at the wheel center.

The schematic diagrams of the wheel wander distribution modes for non-platooning and platooning trucks are shown in Fig. 4-10.

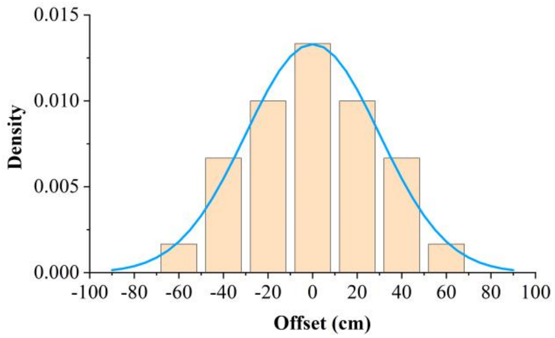


(a)

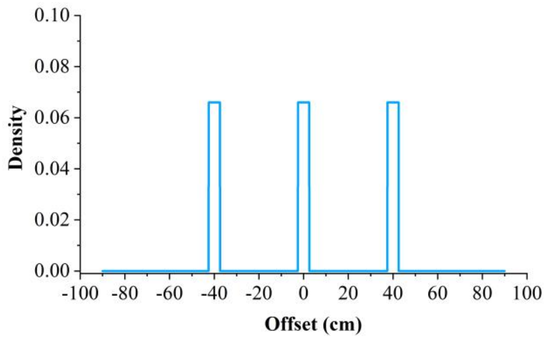


(b)

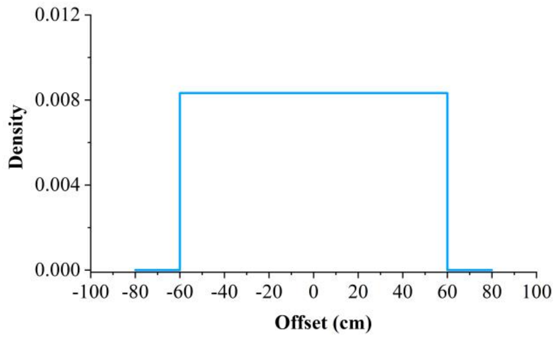


(c)

**Fig. 4- 10. The wheel wander distribution modes for the non-platooning and platooning trucks: (a) the normal distribution mode for non-platooning trucks, (b) the mode 1 for platooning trucks, and (c) the mode 2 for platooning trucks.**

Each platooning mode is further divided into two sub-controlling modes:

- Sub mode 1: All platooning trucks are randomly assigned to follow the specially designed distribution mode.
- Sub mode 2: Trucks within a platoon follow the same driving path, while trucks in different platoons are assigned to follow the specially designed distribution mode.

The schematic diagrams of the two sub modes are presented in Fig. 4-11. It is seen that in sub mode 2, the leading truck in a platoon resists most of the drag resistance, while the trailing ones experience less air resistance. However, in sub mode 1, the trailing trucks may still face noticeable air resistance due to the lateral offset from the driving path of the leading truck.

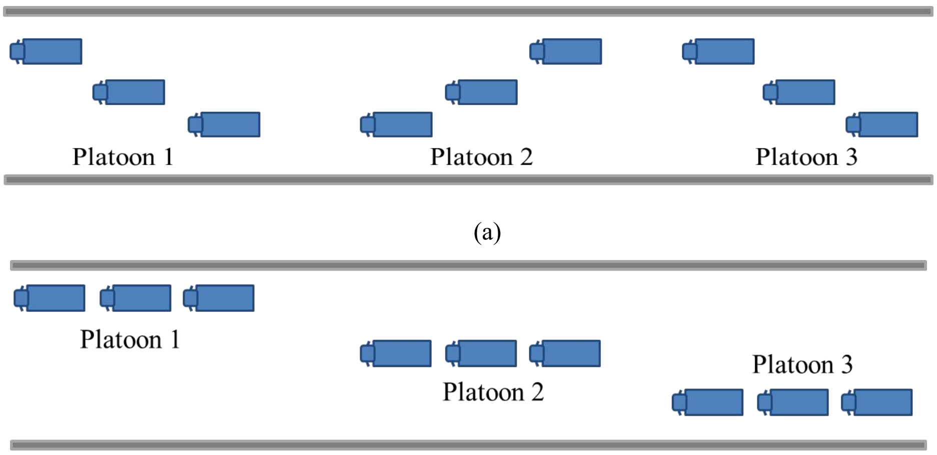


(b)

**Fig. 4- 11. The schematic diagrams of truck distributions under (a) Sub mode 1 and (b) Sub mode 2.**

Based on the distributions described above, the critical strain responses of the road pavement under non-platooning and platooning trucks were calculated. The resulting critical strain values were then used in fatigue life prediction models to estimate the fatigue damage of the road pavement under a specific axle load, along with the shift factor. It was found that the combination Platooning mode 2 + Sub mode 2 is the most beneficial strategy for reducing fatigue damage caused by platooning trucks on the road. This combination is also the best solution for reducing air resistance and fuel consumption of platooning trucks. Therefore, the Platooning mode 2 + Sub mode 2 strategy is ultimately adopted as the wheel wander control method for platooning trucks in this research.

### 4.3 Development of the road damage model

Based on results from Sections 4.1 & 4.2, the damage accumulation model is developed to calculate the cumulative damage of the road pavement under repetitive truck loads. The damage accumulation model has the following form.

$$\begin{cases} D = \sum_{i=1}^{k} \frac{n_i}{N_{f,i}} \\ N_{f,i} = a\left(\frac{1}{\varepsilon_i}\right)^b \cdot (\frac{1}{E_i})^c \cdot e^{(d \cdot T_i + f \cdot RP_i)} \cdot SF_i \end{cases} \tag{4.3}$$

Where, $n_i$ is the total number of load repetitions in the *i*-th block of truck loading, $k$ is the total number of trucks, $N_{f,i}$ is the fatigue life of the material under the *i*-th truck loading.

An example procedure of calculating pavement damage accumulation is illustrated in Fig. 4-12, where the terms S, Ta and Tr refer to the respective single axle, tandem axle and tridem axle, and RP refers to the rest period. Based on this procedure, the damage state evolutions of the road pavement under different traffic modes (normal vs. truck-platooning) are determined to assist in scheduling maintenance or rehabilitation activities and calculating the corresponding emissions.

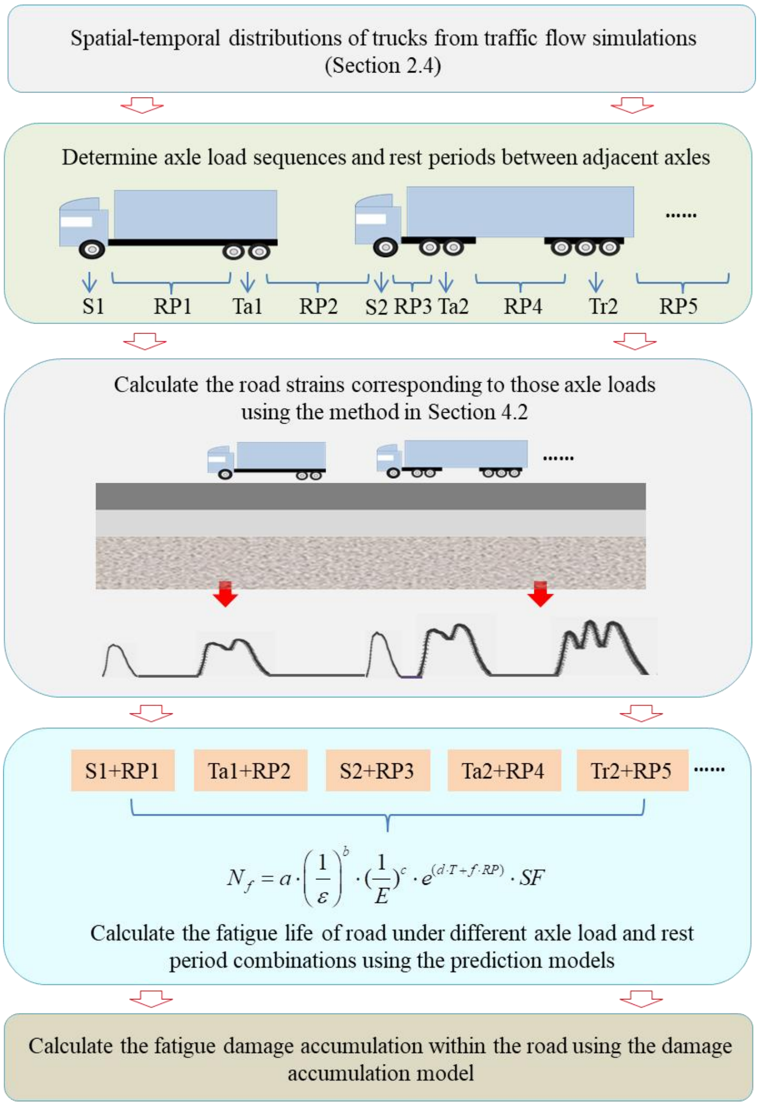


**Fig. 4- 12. The exemplary procedure of calculating the damage amount within the road.**

## Supplementary Method 5. GHG emission and cost model for the integrated vehicle-road infrastructure system

The outputs from Supplementary Methods 1~4 are used to calculate the GHG emissions (costs) of the integrated vehicle-road infrastructure system under normal mode and truck-platooning mode. The GHG emission (cost) models used for calculation are introduced in detail in this chapter.

### 5.1 The overall framework of GHG emission (Cost) models

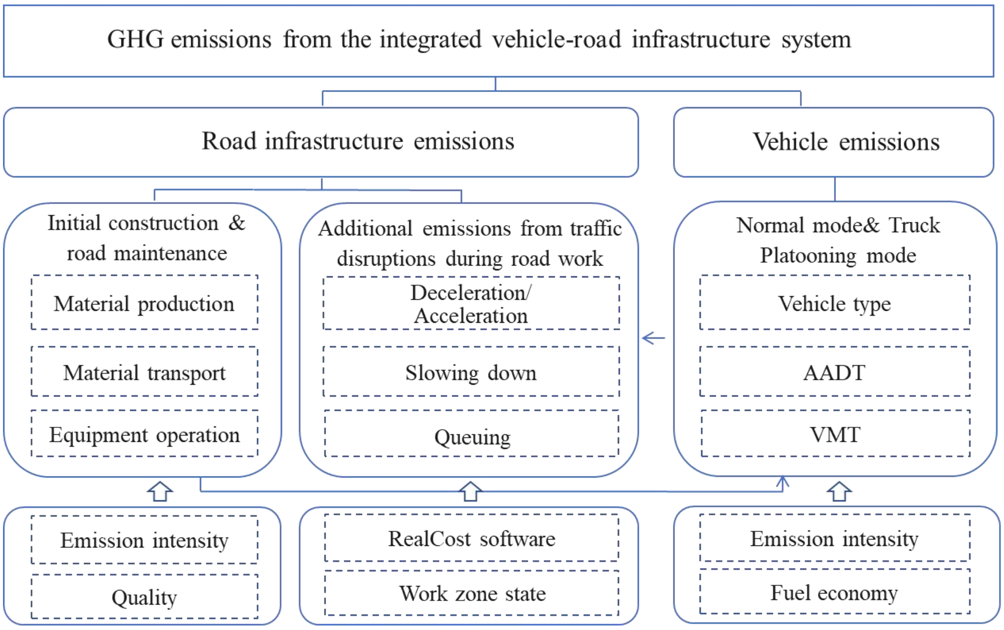


**Fig. 5- 1. The overall framework of the GHG emission model (Note: AADT = average annual daily traffic; VMT =vehicle miles traveled).**

The overall framework of the GHG emission calculation model is presented in Fig. 5-1. As noted, GHG emissions of the integrated system consist of emissions from road infrastructure and vehicles. Emissions from road infrastructure are divided into two portions to facilitate analysis: emissions from the initial construction stage and those from the road maintenance work after construction (i.e., maintenance, rehabilitation and reconstruction). Road emissions at the initial construction stage include emissions from material production, material transport and construction equipment operations. By contrast, road emissions at the maintenance stage are generated from the maintenance material production & transport and maintenance equipment operations. The end-of-life (EOL) processing of road materials (i.e., milling and transport) is also considered in the maintenance stage through transport and equipment operation modules. In addition, traffic disruptions due to lane closure during maintenance work also account for the maintenance stage's emissions. Traffic disruptions, including deceleration, acceleration, slowing down and even queuing of vehicles, produce extra emissions as compared with those generated in normal vehicle operations. Even though such extra emissions are directly from traveling vehicles, they are only generated during road maintenance work and thus are assigned to road infrastructure emissions. The vehicle emissions are attributed to those from vehicles traveling on the road sections, which are estimated based on the vehicles' fuel consumption. The two sources of emissions are closely related. Firstly, vehicle emission is dependent on road performance, such as surface roughness

(defined as the international roughness index, IRI). The high roughness of a road increases vehicle emissions. As a result, the road performance data is incorporated into the vehicle emission model. Secondly, vehicle emission models are used to calculate the additional emissions from road-work-related traffic disruptions.

The cost model for the vehicle-road system is similar to that of the emission model, except that the emission intensity in the framework is replaced with the cost intensity. Hence, the framework of the cost model will not be separately introduced. The detailed calculation models for road infrastructure and vehicle emissions are presented as follows.

## 5.2 GHG emissions from road infrastructure

### 5.2.1 GHG emissions from the initial construction stage and the maintenance stage

The emissions in this module are generated during the initial road construction, the road maintenance and the EOL processing of road materials, including the material production, material transport and equipment operations. The following equation is used to calculate the emissions from road construction and maintenance work.

$$GHG_{CME} = \sum_{i=1}^{n} f_{CMEi} \cdot q_{CMEi} \tag{5.1}$$

Where, $GHG_{CME}$ is the emission from the road construction, road maintenance or EOL processing activity, $f_{CMEi}$ is the unit emission intensity of the *i*-th activity, $q_{CMEi}$ is the quantity of the *i*-th activity.

The quantity of the construction, maintenance or EOL processing activity is calculated based on road dimensions (e.g, pavement thickness, lane width, lane length, maintenance length). The unit emission intensities are are summarized in Table 5- 1.

**Table 5- 1. Unit emission intensities from road construction and maintenance activities.**

| Items | Emission intensities |
|---|---|
| **Material production[1]** | |
| Binders in asphalt concrete layer | 0.480 kg $CO_2$-e/kg |
| Aggregates in asphalt concrete layer | 0.010 kg $CO_2$-e/kg |
| Granular base aggregate | 0.002 kg $CO_2$-e/kg |
| Treated base layer | 0.010 kg $CO_2$-e/kg |
| **Material transport[2]** | |
| Binders in asphalt concrete layer | 0.004 kg $CO_2$-e/kg |
| Aggregates in asphalt concrete layer | 0.006 kg $CO_2$-e/kg |
| Granular base aggregate | 0.004 kg $CO_2$-e/kg |
| Treated base layer | 0.006 kg $CO_2$-e/kg |
| Asphalt mixture after demolition | 0.002 kg $CO_2$-e/kg |
| **Equipment operations for layer construction[3]** | |
| Asphalt concrete layer | 1.741 kg $CO_2$-e/$m^3$ |
| Base layer | 0.024 kg $CO_2$-e/$m^3$ |
| Subgrade | 0.315 kg $CO_2$-e/$m^2$ |

| Equipment operations for maintenance work or EOL processing[4] | |
|---|---|
| Cracking sealing | 0.080 kg $CO_2$-e/m$^2$ |
| Patching | 0.400 kg $CO_2$-e/m$^2$ |
| Milling | 0.287 kg $CO_2$-e/m$^2$ |

1 The emission intensities are determined according to references[49-51].

2 The emission intensities are estimated according to surveys as well as the reference[53]. It is assumed that the transport distances from the binder source to plant, gravel source to plant, and gravel source to the site are 40 km, 80 km and 80 km, respectively. The transport distance from the plant to the site is 40 km. The transport distance from the demolition site to the landfill site is 40 km.

3 The emission intensities are determined according to references[49, 50]. The equipment includes clearing and grubbing equipment, subgrade grader and compactor, granular layer grader and compactor, asphalt paver, and asphalt compactor.

4 The emission intensities are determined according to references[52, 53].

### 5.2.2 Additional GHG emissions from traffic disruptions caused by road maintenance work

Traffic disruptions caused by road maintenance work generate additional vehicle emissions above those of free-flow vehicles. The traffic disruption states include deceleration/acceleration, slowing down, and even queuing of the vehicle. Emissions from traffic disruptions are calculated using the following model.

$$GHG_{TD} = \sum_{j=1}^{m} VMT_{TDj} \cdot f_{TDj} - VMT_{Nj} \cdot f_{Nj} \tag{5.2}$$

Where, $GHG_{TD}$ is the additional emissions caused by traffic disruptions, $VMT_{TDj}$ is the vehicle miles traveled at the $j$-th traffic disruption state (deceleration/acceleration, slowing down, queuing), $f_{TDj}$ is the unit emission intensity at the $j$-th traffic disruption state, $VMT_{Nj}$ is the vehicle miles traveled at a normal traffic state (no maintenance), $f_{Nj}$ is the unit emission intensity at a normal traffic state.

The traffic disruption states on the road during construction work are estimated using the RealCost software[54]. The input parameters for the RealCost are summarized in Table 5- 2.

**Table 5- 2. The input parameters for the RealCost software.**

| Items | Values |
|---|---|
| Hourly traffic data within 24h | Obtained from the LTPP database |
| Percentage of passenger car | |
| Percentage of single truck | |
| Percentage of combo truck | |
| Lanes open in each direction under normal condition[1] | |
| Speed limit under normal operating conditions | 90 km/h |
| Lanes closed during maintenance[1] | 1 |
| Free flow capacity | Predicted using the model[2] |

| | |
|---|---|
| Queue dissipation capacity | Predicted using the model[3] |
| Maximum AADT | Predicted using the model[4] |
| Work zone length | 1 km |
| Work zone operations | For reconstruction (milling & resurfacing): 45 days<br>For overlay: 15 days<br>For sealing and patching: 5 days |
| Work zone capacity | Predicted using the model[5] |
| Work zone speed limit | 40 km/h |
| Work zone hours | The lane is assumed to be closed from 8 a.m. until 18 p.m |

1 It is assumed that one lane is closed in each direction during road maintenance work. If there is only one lane open in each direction under normal conditions, all vehicles will have to detour during road maintenance. Under this condition, the traffic-disruption-related emissions are calculated as the emissions generated during the self-detour of vehicles. The detour distance is assumed as 2.5 km[51].

2 The free flow capacity for the road is predicted using the following model.

$$FFC = \frac{F \cdot 100}{100 + P \cdot (E-1)} \tag{5.3}$$

Where, $FFC$ is the free flow capacity (vehicle per hour per lane, vphpl). $F$ is the roadway capacity (passenger car per hour per lane, pcphpl). 2200 pcphpl is used for 2- or fewer lane highways while 2300 is used for 3- or more lane highways. $P$ is the percentage of heavy vehicles. $E$ is the passenger car equivalent factor, which has a value of 1.5 in this research.

3 The queue dissipation capacity is calculated using the following model.

$$QC = \frac{Q \cdot 100}{100 + P \cdot (E-1)} \tag{5.4}$$

Where, $QC$ is the queue dissipation capacity (vehicle per hour per lane, vphpl). $Q$ is the base capacity (passenger car per hour per lane, pcphpl). 1800 pcphp is used in this research. $P$ is the percentage of heavy vehicles. $E$ is the passenger car equivalent factor (1.5).

4 The maximum AADT is calculated using the following model.

$$AADT_{max} = \frac{M \cdot N \cdot 100}{100 + P \cdot (E-1)} \tag{5.5}$$

Where, $AADT_{max}$ is the maximum AADT (total for both directions). $M$ is 43000 for two-lane highways. $N$ is the number of lanes (total for both directions). $P$ is the percentage of heavy vehicles. $E$ is the passenger car equivalent factor (1.5).

5 The work zone capacity is calculated using the following model.

$$WC = \frac{W \cdot 100}{100 + P \cdot (E-1)} \tag{5.6}$$

Where, $WC$ is the work zone capacity (vehicle per hour per lane, vphpl)). $W$ is the base work zone capacity (passenger car per hour per lane, pcphpl). 1300 pcphl is used for two-lane highways, while 1500 pcphl is used for three- or more lane highways. $P$ is the percentage of heavy vehicles. $E$ is the passenger car equivalent factor (1.5).

Based on the traffic disruption states, additional emissions during road work are estimated by combining the corresponding emission intensity data, which are summarized in Table 5- 3.

**Table 5- 3. Unit emission intensities corresponding to traffic disruption states.**

| Items | Emission intensities |
| --- | --- |
| Deceleration/Acceleration process[1]: From normal speed to work zone speed/From work zone speed to normal speed | Passenger car: 10 g $CO_2$-e/s<br>Single truck: 200 g $CO_2$-e/s<br>Combo truck: 110 g $CO_2$-e/s |
| Deceleration/Acceleration process[1]: From normal speed to queuing speed/From queuing speed to normal speed | Passenger car: 7 g $CO_2$-e/s<br>Single truck: 150 g $CO_2$-e/s<br>Combo truck: 90 g $CO_2$-e/s |
| Slowing down & Queuing | Predicted using the model[2] |

1 The emission intensities are determined according to references [55, 56].

2 The emission intensities of vehicles at the slowing-down state or queuing state are estimated using Equations (5.7)~(5.9), which are developed based on references [50, 57, 58].

$$EI_{car} = 16.42V^{-1} + 0.38 - 4.26\times10^{-3}V + 8.14\times10^{-5}V^2 \quad (5.7)$$

$$EI_{single} = 10.22V^{-1} + 1.49 - 2.20\times10^{-2}V + 1.51\times10^{-4}V^2 \quad (5.8)$$

$$EI_{combo} = 12.80V^{-1} + 1.86 - 2.76\times10^{-2}V + 1.89\times10^{-4}V^2 \quad (5.9)$$

Where, $EI_{car}$ is the emission intensity of the passenger car (kg $CO_2$-e /km), $EI_{single}$ is the emission intensity of the single truck (kg $CO_2$-e /km), $EI_{combo}$ is the emission intensity of the combo truck (kg $CO_2$-e /km), and $V$ is the vehicle speed (km/h) at the slowing-down state or queuing state.

## 5.3 GHG emissions from normal vehicle operations

Vehicle emissions are estimated based on the vehicles' fuel consumption. Two traffic modes namely normal and truck-platooning modes are included in this research. In truck platooning mode, the fuel consumption of trucks is saved due to reductions in air resistance (see Supplementary Method 3 Equation (3.9)). In addition, to consider the effect of road roughness on vehicle emissions, the IRI data is also incorporated into the vehicle emission model. The following model is developed to predict vehicle emissions.

$$GHG_V = \sum_{i=1}^{n} f_{Vi} \cdot VMT_i \cdot FC_i \cdot (1 - \Delta FC_{Pi}) \cdot (1 + \Delta FC_{IRIi} \cdot \Delta IRI) \quad (5.10)$$

Where, $GHG_V$ is the emission from vehicles, $f_{Vi}$ is the unit emission intensity of the *i*-th vehicle. $VMT_i$ is the vehicle miles traveled of the *i*-th vehicle. $FC_i$ is the fuel consumption of the *i*-th vehicle. $\Delta FC_{Pi}$ is the saving rate due to truck platooning. If the target vehicle is a passenger car, $\Delta FC_{Pi}$ is determined as 0%. Otherwise, $\Delta FC_{Pi}$ is calculated using Equation (3.9). $\Delta FC_{IRIi}$ is the variation rates of vehicle fuel consumption due to road's IRI performance. $\Delta IRI$ is the gap between the road's actual IRI and the baseline IRI (1.0m/km is used in this research).

As mentioned before, the fuel consumption ($FC_i$) of a vehicle is determined according to the statistical Motor-Vehicle Travel (VMT) data from FHWA (see Supplementary Method 3 Table 3-3), which represents the actual fuel economy of vehicles in the U.S. The unit emission intensities and the variation rates of fuel consumption due to road roughness ($\Delta FC_{IRI\_i}$) are determined according to previous research, as shown in Table 5- 4.

**Table 5- 4. The parameter values used in vehicle emission calculation.**

| Items | Values |
|---|---|
| Fuel consumption[1] | Passenger car: 0.106 L/km<br>Single truck: 0.313 L/km<br>Combo truck: 0.392 L/km |
| Emission intensities corresponding to fuel consumption[2] | Passenger car: 2.332 kg $CO_2$-e/L<br>Single truck: 2.699 kg $CO_2$-e/L<br>Combo truck: 2.701 kg $CO_2$-e/L |
| Variation rates of fuel consumption due to IRI ($\Delta FC_{IRIi}$)[3] | Passenger car: 0.915%<br>Single truck: 0.305%<br>Combo truck: 0.686% |

1 The fuel consumption of vehicles is determined according to FHWA statistical data [57].

2 The emission intensities corresponding to fuel consumption are determined according to the reference [50, 59].

3 The variation rates of fuel consumption due to IRI are determined according to the reference [60, 61].

Based on the models in Sections 5.2 and 5.3, the total road-related and vehicle-related GHG emissions are calculated, which represent the overall emissions from the integrated vehicle-road system. The calculation procedure of the cost of the vehicle & road system is similar to that of the emission, and hence is not discussed. The cost intensities used in the cost model are summarized in Table 5- 5.

**Table 5- 5. The cost intensities used in the cost analysis.**

| Items | Cost intensities |
|---|---|
| **Material production[1]** | |
| Asphalt layer | 0.083 \$/kg |
| Granular base aggregate | 0.014 \$/kg |
| Treated base layer | 0.017 \$/kg |
| **Material transport[2]** | |
| Binders in asphalt layer | 0.00089 \$/kg |
| Aggregates in asphalt layer | 0.00133 \$/kg |
| Granular base aggregate | 0.00089 \$/kg |
| Treated base layer | 0.00133 \$/kg |
| Asphalt mixture after demolition | 0.00045 \$/kg |
| **Equipment operations for layer construction[3]** | |
| Asphalt concrete layer | 43.0 $\$/m^2$ |
| Base layer | 22.0 $\$/m^2$ |
| Subgrade | 22.0 $\$/m^2$ |

| | |
|---|---|
| **Equipment operations for maintenance work or EOL processing[4]** | |
| Cracking sealing | 125.0 \$/m |
| Patching | 113.6 $\$/m^2$ |
| Milling | 3.6 $\$/m^2$ |
| **Traffic disruption** | |
| Time delay costs[1] | Passenger car: 28.70 \$/h<br>Single truck: 22.42 \$/h<br>Combo truck: 29.27 \$/h |
| Deceleration/Acceleration process[5]:<br>From normal speed to work zone speed/From work zone speed to normal speed | Passenger car: 0.0773 \$/vehicle<br>Single truck: 0.1365 \$/vehicle<br>Combo truck: 0.6560 \$/vehicle |
| Deceleration/Acceleration process[5]:<br>From normal speed to queuing speed/From queuing speed to normal speed | Passenger car: 0.1177 \$/vehicle<br>Single truck: 0.2268 \$/vehicle<br>Combo truck: 1.0177 \$/vehicle |
| Queuing (Vehicle idling cost)[5] | Passenger car: 0.9767 \$/vehicle/h<br>Single truck: 1.0830 \$/vehicle/h<br>Combo truck: 1.1630 \$/vehicle/h |
| Slowing down & Queuing (Additional fuel consumption cost)[6] | Predicted using the model[2] |
| **Vehicle operation[3]** | |
| Cost intensities corresponding to fuel consumption | Gasoline: 0.53 \$/L<br>Diesel: 0.63 \$/L |

1 The cost intensities corresponding to material production and time delay are determined according to the reference[62].
2 The cost intensities corresponding to material transport are determined according to references [51, 63].
3 The cost intensities corresponding to material processing and fuel consumption are determined according to the reference[63].
4 The cost intensities corresponding to maintenance work are determined according to the reference[64].
5 The costs during the deceleration/acceleration and queuing process are determined according to references[56, 64].

6 The cost intensities of vehicles at the slowing-down state or queuing state are estimated using Equations (5.11) ~(5.13), which are developed based on references [57, 58, 63].

$$CI_{car} = 1.10V^{-1} + 0.02 - 2.85\times10^{-4}V + 5.44\times10^{-6}V^2 \tag{5.11}$$

$$CI_{single} = 2.38V^{-1} + 0.35 - 5.10\times10^{-3}V + 3.52\times10^{-5}V^2 \tag{5.12}$$

$$CI_{combo} = 2.98V^{-1} + 0.43 - 6.42\times10^{-3}V + 4.41\times10^{-5}V^2 \tag{5.13}$$

Where, $CI_{car}$ is the cost intensity of the passenger car (\$/km), $CI_{single}$ is the cost intensity of the single truck (\$/km), $CI_{combo}$ is the cost intensity of the combo truck (\$/km), and $V$ is the vehicle speed (km/h) at the slowing-down state or queuing state.

In addition to the cost components shown in Table 5-5, drivers also have to bear the costs of tire wear-and-tear (W&T) and vehicle repair-and-maintenance (R&M). Both these costs depend on the pavement surface condition (i.e., IRI). A better pavement condition generally leads to lower costs. To calculate the W&T and R&M costs, Equations (5.14) and (5.15) were developed according to the data shown in the literature[65].

$$Cost_{W\&T} = CI_{W\&T} \cdot [1 + C_{W\&T}(IRI)] \tag{5.14}$$

$$Cost_{R\&M} = CI_{R\&M} \cdot [1 + C_{R\&M}(IRI)] \tag{5.15}$$

Where, $CI_{W\&T}$ and $CI_{R\&M}$ refer to the intensity of two types of cost, and $C_{W\&T}(IRI)$ and $C_{R\&M}(IRI)$ refer to the adjusting functions related to the pavement's IRI. The exact intensity values and adjusting functions for different vehicles are summarized in Table 5-6.

**Table 5- 6. The intensity values and adjusting functions for different vehicles.**

| **Items** | Passenger car | Single truck | Combo truck |
|---|---|---|---|
| $CI_{W\&T}$ | 0.0015 \$/km/tire | 0.0056 \$/km/tire | 0.007 \$/km/tire |
| $CI_{R\&M}$ | 0.04 \$/km | 0.06 \$/km | 0.12 \$/km |
| $C_{W\&T}(IRI)$ [1] | $0.0018 \cdot IRI^{2.34}$ | $0.0020 \cdot IRI^{2.16}$ | $0.0018 \cdot IRI^{2.31}$ |
| $C_{R\&M}(IRI)$ [1] | $5.1 \times 10^{-5} \cdot e^{1.93 \cdot IRI}$ | $1.3 \times 10^{-4} \cdot e^{1.74 \cdot IRI}$ | $1.1 \times 10^{-4} \cdot e^{1.91 \cdot IRI}$ |

1 The unit of the IRI parameter in equations is m/km.

## Supplementary references


[1] FHWA, The Long-Term Pavement Performance Program, Federal Highway Administration, McLean, VA, (2015).

[2] G.E. Elkins, B. Ostrom, Long-term pavement performance information management system user guide, FHWA Office of Infrastructure Research and Development. (2021).

[3] Y. Wang, Development of a pavement climate map based on LTPP data, 2001-2002 International Contest on Long-Term Pavement Performance Data Analysis. 111-137 (2005).

[4] Y. Wang, G. Wang, Y.H. Ahn, Impact of climate conditions on effectiveness of asphalt pavement preservation techniques, Transportation Research Record. 2292(1), 73-80 (2012).

[5] Dimensions, https://www.dimensions.com. (2021).

[6] 360che, http://www.360che.com/. (2021).

[7] Kargo, https://kargo.tech. (2021).

[8] M. Treiber, A. Hennecke, D. Helbing, Congested traffic states in empirical observations and microscopic simulations, Physical review E. 62(2), 1805 (2000).

[9] F. Ye, Y. Zhang, Vehicle type–specific headway analysis using freeway traffic data, Transportation Research Record. 2124(1), 222-230 (2009).

[10] M. Treiber, A. Kesting, Traffic flow dynamics, Springer, London, 2013.

[11] L. Liu, L. Zhu, D. Yang, Modeling and simulation of the car-truck heterogeneous traffic flow based on a nonlinear car-following model, Applied Mathematics and Computation. 273, 706-717 (2016).

[12] V. Milanés, S.E. Shladover, Handling cut-in vehicles in strings of cooperative adaptive cruise control vehicles, Journal of Intelligent Transportation Systems. 20(2), 178-191 (2016).

[13] L. Xiao, M. Wang, B. Van Arem, Realistic car-following models for microscopic simulation of adaptive and cooperative adaptive cruise control vehicles, Transportation Research Record. 2623(1), 1-9 (2017).

[14] V. Milanés, S.E. Shladover, Modeling cooperative and autonomous adaptive cruise control dynamic responses using experimental data, Transportation Research Part C: Emerging Technologies. 48, 285-300 (2014).

[15] H. Liu, X. Kan, S.E. Shladover, X.-Y. Lu, R.E. Ferlis, Impact of cooperative adaptive cruise control on multilane freeway merge capacity, Journal of Intelligent Transportation Systems. 22(3), 263-275 (2018).

[16] L. Xiao, M. Wang, W. Schakel, B. van Arem, Unravelling effects of cooperative adaptive cruise control deactivation on traffic flow characteristics at merging bottlenecks, Transportation Research Part C: Emerging Technologies. 96, 380-397 (2018).

[17] A.K. Bhoopalam, N. Agatz, R. Zuidwijk, Planning of truck platoons: A literature review and directions for future research, Transportation Research Part B: Methodological. 107, 212-228 (2018).

[18] J. Smith, R. Mihelic, B. Gifford, M. Ellis, Aerodynamic impact of tractor-trailer in drafting configuration, SAE International Journal of Commercial Vehicles. 7(2), 619-625 (2014).

[19] V. Vohra, M. Wahba, G. Akarslan, R. Ni, S. Brennan, An examination of vehicle spacing to reduce aerodynamic drag in truck platoons, 2018 IEEE Vehicle Power and Propulsion Conference (VPPC). 1-6 (2018).

[20] H.L. Humphreys, J. Batterson, D. Bevly, R. Schubert, An evaluation of the fuel economy benefits of a driver assistive truck platooning prototype using simulation, SAE Technical Paper 2016-01-0167, 1-9 (2016).

[21] B. McAuliffe, M. Croken, M. Ahmadi-Baloutaki, A. Raeesi, Fuel-economy testing of a three-vehicle truck platooning system, Laboratory Technical Report, National Research Council, Canada. LTR-AL-2017-0008 (2017).

[22] S. Tsugawa, S. Jeschke, S.E. Shladover, A review of truck platooning projects for energy savings, IEEE Transactions on Intelligent Vehicles. 1(1), 68-77(2016).

[23] S. Tsugawa, S. Kato, K. Aoki, An automated truck platoon for energy saving, 2011 IEEE/RSJ International Conference on Intelligent Robots and Systems, 4109-4114 (2011).
[24] C. Xia, H. Wang, X. Shan, Z. Yang, Q. Li, Effects of ground configurations on the slipstream and near wake of a high-speed train, Journal of Wind Engineering and Industrial Aerodynamics, 168, 177-189 (2017).
[25] M. Gophane, G. Salvi, G. Pratik, K. Ravi, Effect of aerodynamic forces over the bus body and design of conceptual bus for enhanced performance, International Journal of Engineering Trends and Technology. 11(4), 159-162 (2014).
[26] M. Hammache, M. Michaelian, F. Browand, Aerodynamic forces on truck models, including two trucks in tandem, California PATH Research Report, UCB-ITS-PRR-2001-27. (2001).
[27] M. Song, F. Chen, X. Ma, Organization of autonomous truck platoon considering energy saving and pavement fatigue, Transportation Research Part D: Transport and Environment. 90, 102667 (2021).
[28] B. McAuliffe, M. Lammert, X.-Y. Lu, S. Shladover, M.-D. Surcel, A. Kailas, Influences on energy savings of heavy trucks using cooperative adaptive cruise control, SAE Technical Paper 2018-01-1181, 1-20 (2018).
[29] SAE J1321, Fuel Consumption Test Procedure - Type II, Surface Vehicle Recommended Practice J1321. (2012).
[30] FHWA, Annual vehicle distance traveled in miles and related data, https://www.fhwa.dot.gov/policyinformation/statistics/2019/vm1.cfm. (2019).
[31] A. Bhasin, D.N. Little, R. Bommavaram, K. Vasconcelos, A framework to quantify the effect of healing in bituminous materials using material properties, Road Materials and Pavement Design. 9(sup1), 219-242 (2008).
[32] H.-J. Lee, Y.R. Kim, Viscoelastic continuum damage model of asphalt concrete with healing, Journal of Engineering Mechanics. 124(11), 1224-1232 (1998).
[33] D. Little, R. Lytton, D. Williams, C. Chen, Microdamage healing in asphalt and asphalt concrete, Volume 1: microdamage and microdamage healing, project summary report, Turner-Fairbank Highway Research Center, (2001).
[34] W.A. Zeiada, P.P. Gudipudi, B.S. Underwood, M.I. Souliman, Effect of loading waveform pattern and rest period on fatigue life of asphalt concrete using viscoelastic continuum damage model, Transportation Research Record. 2672(28), 451-461 (2018).
[35] M. Witczak, M. Mamlouk, M. Souliman, W. Zeiada, Laboratory validation of an endurance limit for asphalt pavements, NCHRP Report 762, Transportation Research Board, (2013).
[36] W.A. Zeiada, M.I. Souliman, K.E. Kaloush, M. Mamlouk, B.S. Underwood, Comparison of fatigue damage, healing, and endurance limit with beam and uniaxial fatigue tests, Transportation Research Record. 2447(1), 32-41 (2014).
[37] S.H. Carpenter, S. Shen, Dissipated energy approach to study hot-mix asphalt healing in fatigue, Transportation Research Record. 1970(1), 178-185 (2006).
[38] J.S. Daniel, Y.R. Kim, Laboratory evaluation of fatigue damage and healing of asphalt mixtures, Journal of Materials in Civil Engineering. 13(6), 434-440 (2001).
[39] M. Souliman, Integrated predictive model for healing and fatigue endurance limit for asphalt concrete, Doctor Thesis, Arizona State University. (2012).
[40] Y.R. Kim, M.N. Guddati, B.S. Underwood, T. Yun, V. Subramanian, S. Savadatti, Development of a multiaxial viscoelastoplastic continuum damage model for asphalt mixtures, FHWA-HRT-08-073, Turner-Fairbank Highway Research Center, (2009).
[41] M.E. Kutay, M. Lanotte, Viscoelastic continuum damage (VECD) models for cracking problems in asphalt mixtures, International Journal of Pavement Engineering. 19(3), 231-242 (2018).
[42] H.-J. Lee, Y.R. Kim, Viscoelastic constitutive model for asphalt concrete under cyclic loading, Journal of Engineering Mechanics. 124(1), 32-40 (1998).
[43] B.S. Underwood, Y.R. Kim, M.N. Guddati, Improved calculation method of damage parameter in

viscoelastic continuum damage model, International Journal of Pavement Engineering. 11(6), 459-476 (2010).
[44] Z. Ma, L. Liu, Y. Yuan, L. Sun, Estimation of total fatigue life for in-service asphalt mixture based on accelerated pavement testing and four-point bending beam fatigue tests, Canadian Journal of Civil Engineering. 46(7), 557-566 (2019).
[45] H. Cheng, L. Sun, R. Yang, Y. Zhang, L. Liu, Relating field moduli of asphalt mixture layer under vehicular loading and its dynamic moduli under laboratory loading, Transportation Research Record, 2676(2), 567-579 (2022).
[46] NCHRP, Guide for mechanistic-empirical design of new and rehabilitated pavement structures, Final Report for Project 1-37A, Transportation Research Board. (2004).
[47] E. Okte, I.L. Al-Qadi, Impact of autonomous and human-driven trucks on flexible pavement design, Transportation Research Record, 2676(7), 144-160 (2022).
[48] F. Chen, M. Song, X. Ma, A lateral control scheme of autonomous vehicles considering pavement sustainability, Journal of Cleaner Production, 256, 120669 (2020).
[49] World Bank, ROADEO (Road Emissions Optimization) software, (2011).
[50] D. Chong, Y. Wang, Impacts of flexible pavement design and management decisions on life cycle energy consumption and carbon footprint, The International Journal of Life Cycle Assessment. 22(6), 952-971 (2017).
[51] H. Zhang, M.D. Lepech, G.A. Keoleian, S. Qian, V.C. Li, Dynamic life-cycle modeling of pavement overlay systems: Capturing the impacts of users, construction, and roadway deterioration, Journal of Infrastructure Systems. 16(4), 299-309 (2010).
[52] J. Chehovits, L. Galehouse, Energy usage and greenhouse gas emissions of pavement preservation processes for asphalt concrete pavements, Proceedings on the 1st International Conference of Pavement Preservation, 27-42 (2010).
[53] X. Chen, H. Wang, R. Horton, J. DeFlorio, Life-cycle assessment of climate change impact on time-dependent carbon-footprint of asphalt pavement, Transportation Research Part D: Transport and Environment. 91, 102697 (2021).
[54] FHWA, RealCost software (Version 2.5): life-cycle cost analysis software product, http://www.fhwa.dot.gov/infrastructure/asstmgmt/lccasoft.cfm. (2011).
[55] K. Zhang, S. Batterman, F. Dion, Vehicle emissions in congestion: Comparison of work zone, rush hour and free-flow conditions, Atmospheric Environment. 45(11), 1929-1939 (2011).
[56] J. Walls, M.R. Smith, Life-cycle cost analysis in pavement design: Interim technical bulletin, FHWA-SA-98-079, Federal Highway Administration. (1998).
[57] FHWA Highway Statistics 2020. https://www.fhwa.dot.gov/policyinformation/statistics/2020/hm220.cfm, (2020).
[57] Y. Song, E. Yao, T. Zuo, Z. Lang, Emissions and fuel consumption modeling for evaluating environmental effectiveness of ITS strategies, Discrete Dynamics in Nature and Society. 2013, 581945 (2013).
[59] EPA, Motor Vehicle Emission Simulator (MOVES) software, http://www.epa.gov/otaq/models/moves. (2014).
[60] K. Chatti, I. Zaabar, Estimating the effects of pavement condition on vehicle operating costs, NCHRP Report 720, Transportation Research Board. (2012).
[61] R. Yang, Development of a pavement life cycle assessment tool utilizing regional data and introducing an asphalt binder model, Doctor Thesis, University of Illinois at Urbana-Champaign. (2014).
[62] J. Santos, J. Bryce, G. Flintsch, A. Ferreira, A comprehensive life cycle costs analysis of in-place recycling and conventional pavement construction and maintenance practices, International Journal of Pavement Engineering. 18(8), 727-743 (2017).
[63] Y. Qiao, E. Dave, T. Parry, O. Valle, L. Mi, G. Ni, Z. Yuan, Y. Zhu, Life cycle costs analysis of reclaimed asphalt pavement (RAP) under future climate, Sustainability. 11(19), 5414 (2019).

[64] T. Rehan, Y. Qi, A. Werner, Life-cycle cost analysis for traditional and permeable pavements, Construction Research Congress, 2018, 422-431 (2018).
[65] E. Okte, I L. Al-Qadi, H. Ozer, Effects of pavement condition on LCCA user costs. Transportation Research Record, 2673(5), 339-350 (2019).
