# Supplementary material for: Truck platooning reshapes greenhouse gas emissions of the integrated vehicle-road infrastructure system
Source: Nat Commun. 2023 Aug 15;14:4495. doi: 10.1038/s41467-023-40116-0 (PMC10427667; doi:10.1038/s41467-023-40116-0)
Supplement: Supplementary file 3 — Description of Additional Supplementary Files [file 41467_2023_40116_MOESM3_ESM.pdf]

Description of Additional Supplementary Files

File Name: Supplementary Data 1
Description: Basic information of road sections

File Name: Supplementary Data 2
Description: IRI data of road sections

File Name: Supplementary Data 3
Description: Maintenance data of road sections

File Name: Supplementary Data 4
Description: Structure and material data of road sections

File Name: Supplementary Data 5
Description: Traffic data of road sections
